# Exercise-induced pain changes associate with changes in muscle perfusion in knee osteoarthritis: exploratory outcome analyses of a randomised controlled trial

**DOI:** 10.1186/s12891-019-2858-8

**Published:** 2019-10-27

**Authors:** Elisabeth Bandak, Mikael Boesen, Henning Bliddal, Robert G. C. Riis, Sabrina Mai Nielsen, Louise Klokker, Cecilie Bartholdy, Janus Damm Nybing, Marius Henriksen

**Affiliations:** 1 The Parker Institute, Copenhagen University Hospital, Bispebjerg and Frederiksberg, Copenhagen, Denmark; 2 Department of Radiology, Copenhagen University Hospital, Bispebjerg and Frederiksberg, Copenhagen, Denmark; 3 Research Unit of Rheumatology, Department of Clinical Research, University of Southern Denmark, Odense University Hospital, Odense, Denmark; 4 Department of physical and occupational therapy, Copenhagen University Hospital, Bispebjerg and Frederiksberg, Copenhagen, Denmark

**Keywords:** Muscle perfusion, Exercise, Pain, Osteoarthritis

## Abstract

**Background:**

Exercise therapy is recommended for knee osteoarthritis (OA), but the underlying mechanisms of pain relief are not fully understood. The purpose of this study was to explore the effects of exercise on muscle perfusion assessed by dynamic contrast enhanced MRI (DCE-MRI) and its association with changes in pain in patients with knee OA.

**Methods:**

Exploratory outcome analyses of a randomised controlled study with per-protocol analyses (ClinicalTrials.gov: NCT01545258) performed at an outpatient clinic at a public hospital in Denmark. We compared 12 weeks of supervised exercise therapy 3 times per week (ET) with a no attention control group (CG). Analyses of covariance (ANCOVA) were used to assess group mean differences in changes from baseline to week 12 in knee muscle perfusion quantified by DCE-MRI, patient-reported pain and function using the Knee Injury and Osteoarthritis Outcome Score (KOOS) questionnaire, knee extensor and flexor muscle strength tests, and the six-minute walking test (6MWT). Spearman’s correlation coefficients were used to determine the correlation between changes in DCE-MRI variables, KOOS, muscle strength, and 6MWT. The potential effect mediation of the DCE-MRI perfusion variables was investigated in a post-hoc mediation analysis.

**Results:**

Of 60 participants randomised with knee osteoarthritis, 33 (ET, *n* = 16, CG, *n* = 17) adhered to the protocol and had complete DCE-MRI data**.** At follow-up, there were significant group differences in muscle perfusion changes and clinically relevant group differences in KOOS pain changes (10.7, 95% CI 3.3 to 18.1, *P* = 0.006) in favor of ET. There were no significant between-group differences on muscle strength and function. The changes in pain and muscle perfusion were significantly correlated (highest Spearman’s rho = 0.42, *P* = 0.014). The mediation analyses were generally not statistically significant.

**Conclusion:**

The pain-reducing effects of a 12-week exercise program are associated with changes in knee muscle perfusion quantified by DCE-MRI in individuals with knee OA, but whether the effects are mediated by muscle perfusion changes remains unclear.

**Trial registration:**

ClinicalTrials.gov: NCT01545258, first posted March 6, 2012.

## Background

Exercise therapy is recommended as first line treatment of knee osteoarthritis (OA) based on extensive research evidence on beneficial effects of exercise on pain and function [1–4]. However further investigations of the underlying mechanisms of exercise are needed in order to substantiate a possible mode of action.

The beneficial effects of exercise on knee OA pain and function may be caused by increased muscle strength, increased knee range of motion, or improvements in proprioception [5, 6]. Also, a reduction of pressure-pain sensitivity in parallel with pain reduction has indicated alterations in pain processing as a mechanism of exercise [7]. The measurement instruments for these outcomes, however, are either performance-based or assessor-dependent, which lowers the objectivity of the measure and increases the risk of bias [8]. Objective measurements of local physiological changes are needed in order to understand the mode of action behind the symptomatic relief caused by exercise therapy in knee OA.

Muscles are the primary tissue affected by most exercise types; thus, any physiological changes caused by exercise are expected to be reflected in the muscles. Indeed, exercise causes physiological changes in the muscles, such as increased capillarisation or angiogenesis [9–11]. We have recently shown that more widespread muscle perfusion in the peri-articular knee muscles was associated with less pain in patients with knee OA [12]. However, no conclusions on causality between exercise-induced changes in muscle perfusion and decreased knee OA pain can be drawn based on this cross-sectional material; the association must be tested prospectively.

Dynamic Contrast Enhanced Magnetic Resonance Imaging (DCE-MRI) is a method to quantify tissue perfusion through temporal variations of the MRI signal intensity following intravenous injection of a contrast agent [13]. Such quantification of localized perfusion of synovium reflects the histological degree of inflammation of the synovium in both rheumatoid arthritis and end-stage knee OA [14, 15]. Furthermore, in knee OA, increased perfusion of the infrapatellar fat pad and synovium is associated with higher pain scores [16–18]. DCE-MRI derived perfusion is a sensitive method to assess skeletal muscle perfusion [19].

The purposes of this study were to explore 1) the effects of a 12-week therapeutic exercise program on muscle perfusion in the peri-articular knee muscles assessed by DCE-MRI and 2) the association between changes in muscle perfusion and changes in pain, physical function and performance in patients with knee OA. We hypothesized that exercise would increase muscle perfusion in parallel with improved pain, physical function and performance when compared with a no-attention control intervention.

## Methods

### Study design

This is an exploratory sub-study of a previously published randomised controlled parallel-group-trial investigating the effects of a 12-week exercise program on the pain sensitivity in patients with knee OA focusing on the per-protocol population [7] performed at an outpatient’s clinic at a public hospital in Denmark (2012–2013). The study was approved by the Regional Health Research Ethics Committee of The Capital Region of Denmark (H-2-2011-159) and registered prior to commencement of the trial (www.ClinicalTrials.gov: NCT01545258).

### Participants

A comprehensive description of the recruitment process and inclusion/exclusion criteria is published elsewhere [7]. Briefly, eligibility criteria included age ≥ 40 years, a clinical diagnosis of knee OA confirmed by radiography assessed by an experienced radiologist, and a body mass index between 20 and 35 kg/m^2^. Exclusion criteria included participation in exercise therapy within the previous 3 months, having inflammatory and autoimmune diseases, and lower extremity joint replacement.

### Sample size, randomisation and blinding

The sample size of the main trial was based on group differences in the primary outcome, pressure-pain threshold, and was calculated to 60 participants [7].

After baseline assessments, participants were randomly assigned (1:1 stratified by gender) to exercise therapy (ET) or to a control group (CG). A computer-generated list of random numbers was used and concealed from the researchers enrolling and assessing participants. Participants were aware of their group allocation; outcome assessors and data analysts were kept blinded to the allocation [7].

### Interventions

The ET group was offered facility-based, functional and individualised exercise therapy supervised by a trained physiotherapist 3 times weekly for 12 weeks. A full description of the.

exercise program is available in [7] and in the Additional file 1. Attendance at minimum 24/36 sessions was defined as protocol adherence. The CG received no attention during the 12 weeks and was requested not to engage in therapeutic exercise during study participation.

### MRI protocol

MRI of the most symptomatic knee at baseline was performed on a 3 T Siemens Verio® (3 T Magnetom Verio, Siemens, Erlangen, Germany) system using a 15-channel dedicated send/receive coil. In addition to a standard clinical MRI protocol, a sagittal DCE T1 VIBE sequence was performed with a temporal resolution of 9 s and 30 repetitions. During the third repetition an intravenous injection of 0.1 ml/kg body weight Gadolinium (Gd) contrast agent (Prohance®, Bracco Diagnostics Inc., Italy) was administered (2 ml/s). The total scanning time was 30 min with the DCE-MRI sequence performed after 20 min. The full MRI protocol is available in the Additional file 1.

### Image analysis

**Fig. 1:**
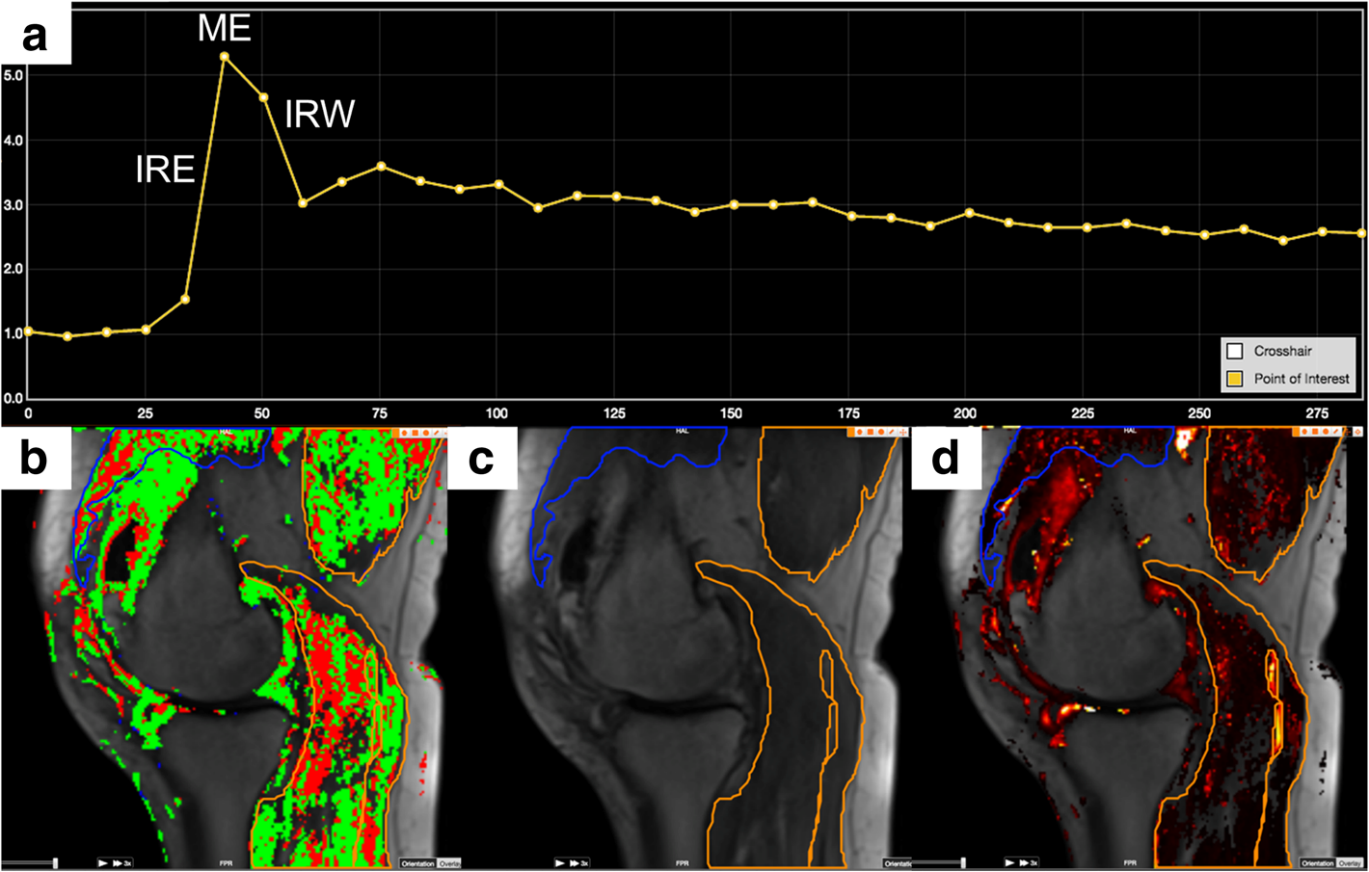
DCE-MRI analysis. DCE-MRI analysis is based on changes in signal intensity over time for each voxel within the VOI. The signal intensity changes calculated relatively to the baseline signal intensity and the changes over time can be plotted as time-intensity-curves (TICs): **a** TIC from a point of interest in the popliteal artery with the characteristics of tissue with high perfusion: rapid increase in signal intensity, which reaches a plateau and a subsequent rapid decrease (washout) (x-axis: time (s); y-axis: relative signal intensity (baseline = 1.0)). IRE: Initial Rate of Enhancement; ME: Maximal Enhancement; IRW: initial rate of washout. **b** Assignment and color–coding of the voxels within the ROIs based on the shape of the TICs: No enhancement (no color); Persistent (blue); Plateau (green) and Washout (red) represent the most perfused voxels. **c** Region of interests (ROIs) around the peri-articular knee muscles avoiding major vascular branches. Blue: extensor muscle; Yellow: flexor muscles. **d** Parametric map of Initial Rate of Enhancement. Brighter colors indicate higher values

The investigator analysing the DCE-MRI images (EB) was blinded to group allocation and clinical data and supervised by experienced radiologists (MB and RR). Dedicated software (Dynamika® version 4.2.2., Image Analysis Ltd., London, UK) was used for all DCE-MRI analyses [20, 21]: motion correction between temporal slices was applied, followed by determination of a baseline level of signal intensity (Fig. 1a), and regions of interests (ROIs) were manually drawn on all slices around the peri-articular muscle groups: extensors (vastus lateralis, vastus medialis) and flexors (biceps femoris, sartorius, gracilis, triceps surae, popliteus, semitendinosus, and semimembranosus) without delineation of individual muscles (Fig. 1c). Major vascular branches were avoided. Finally, the ROIs were summed and averaged into three volumes of interest (VOIs): Extensor VOI (extensor ROIs), Flexor VOI (flexor ROIs) and Total Muscle VOI (consisting of all ROIs).

As previously described in detail [15, 21], heuristic DCE-MRI analysis is based on changes in signal intensity over time in each voxel. The signal intensity changes are calculated relatively to the baseline signal intensity and changes over time can be plotted as time-intensity-curves (TICs) (Fig. 1a). Based on a robust classification scheme [21], the dedicated software automatically assigns each voxel, based on the shape of linear approximations of the TICs, to one of four enhancement patterns (Fig. 1b). The muscle perfusion parameters (see Table 1 and Additional file 1) were calculated on a voxel-by-voxel basis and averaged across each VOI [21, 22]. A pharmacokinetic parameter, K^trans^, was calculated as described in Riis et al. 2016 [16] with the T1-relaxivity values of muscle and blood set at 1420 and 1400 ms, respectively.

**Table 1:** Definitions of the muscle perfusion variables (DCE-MRI)

Variable name	Abbreviation	Definition
Highly Perfused Voxels	Nvoxel	The number of voxels with “plateau” or “washout” enhancement patterns, i.e. the highest perfused voxels, the most perfused tissue.
Proportion of Highly Perfused Voxels (%)	Nvoxel%	The proportion of highly perfused voxels reaching either a “plateau” or “washout” enhancement pattern (Nvoxel) in percentage of the total number of voxels within the VOI.
Initial Rate of Enhancement	IRE	The upslope on the Time intensity curve measured as the mean relative increase in signal intensity per second from enhancement onset until ME is reached(%/s). A surrogate of the degree of perfusion.
Maximal Enhancement	ME	The highest mean signal intensity value relative to the baseline intensity. A surrogate of the degree of perfusion.
Initial Rate of Enhancement Composite Score	IRExNvoxel	The mean relative increase in signal intensity per second (%/s) (IRE) multiplied by the number of highly perfused voxels (Nvoxel). The variable becomes a composite parameter reflecting both the volume (voxels) and the degree of perfusion.
Initial Rate of Enhancement Index	IRExNvoxel%	The mean relative increase in signal intensity per second (%/s) (IRE) multiplied by the proportion of highly perfused voxels (Nvoxel%).
Maximal Enhancement Composite Score	MExNvoxel	The highest mean signal intensity value relative to the baseline intensity (ME) multiplied by the number Highly Perfused Voxels. The variable becomes a composite parameter reflecting both the volume (voxels) and the degree of perfusion.
Maximal Enhancement index	MExNvoxel%	The highest mean signal intensity value relative to the baseline intensity (ME) multiplied by the Proportion of Highly Perfused Voxels.

### Outcomes

Outcomes were measured at baseline and after the 12 weeks of intervention. For the purpose of this exploratory outcome analysis, we examined changes from baseline to follow-up, looking at the outcomes: muscle perfusion from DCE-MRI, patient-reported pain and function using the Knee Injury and Osteoarthritis Outcome Score (KOOS) questionnaire (0(worst)–100(best) [23], physical performance by knee extensor and flexor muscle strength test [24] and the six-minute walking test (6MWT) [25] (see the Additional file 1 for details).

DCE-MRI derived perfusion variables are listed and explained in Table 1. All have previously been used in knee OA- and rheumatoid arthritis studies [14–17, 26, 27]; except Initial Rate of Enhancement and Maximal Enhancement Indices (for details see the Additional file 1).

Intra- and inter observer reproducibility of the DCE-MRI analyses were assessed for the Total VOI on a random subsample (*n* = 10) in which manual segmentations were repeated after a minimum of 4 weeks. The intra-observer reliability of the volume (cm^3^) of the Total Muscle VOI was ICC = 0.99 (95% CI: 0.98 to 0.99). ICC of the muscle perfusion variables was ≥0.99 (in each case, the lower limit of the 95% CI was ≥0.91′). Due to low reproducibility of the K^trans^ it was not included in the further analysis. Details of reproducibility of the DCE-MRI variables are presented in the Additional file 1.

The reliability of KOOS [28], muscle strength tests [24], and 6MWT has been demonstrated [29].

### Statistical methods

**Fig. 2:**
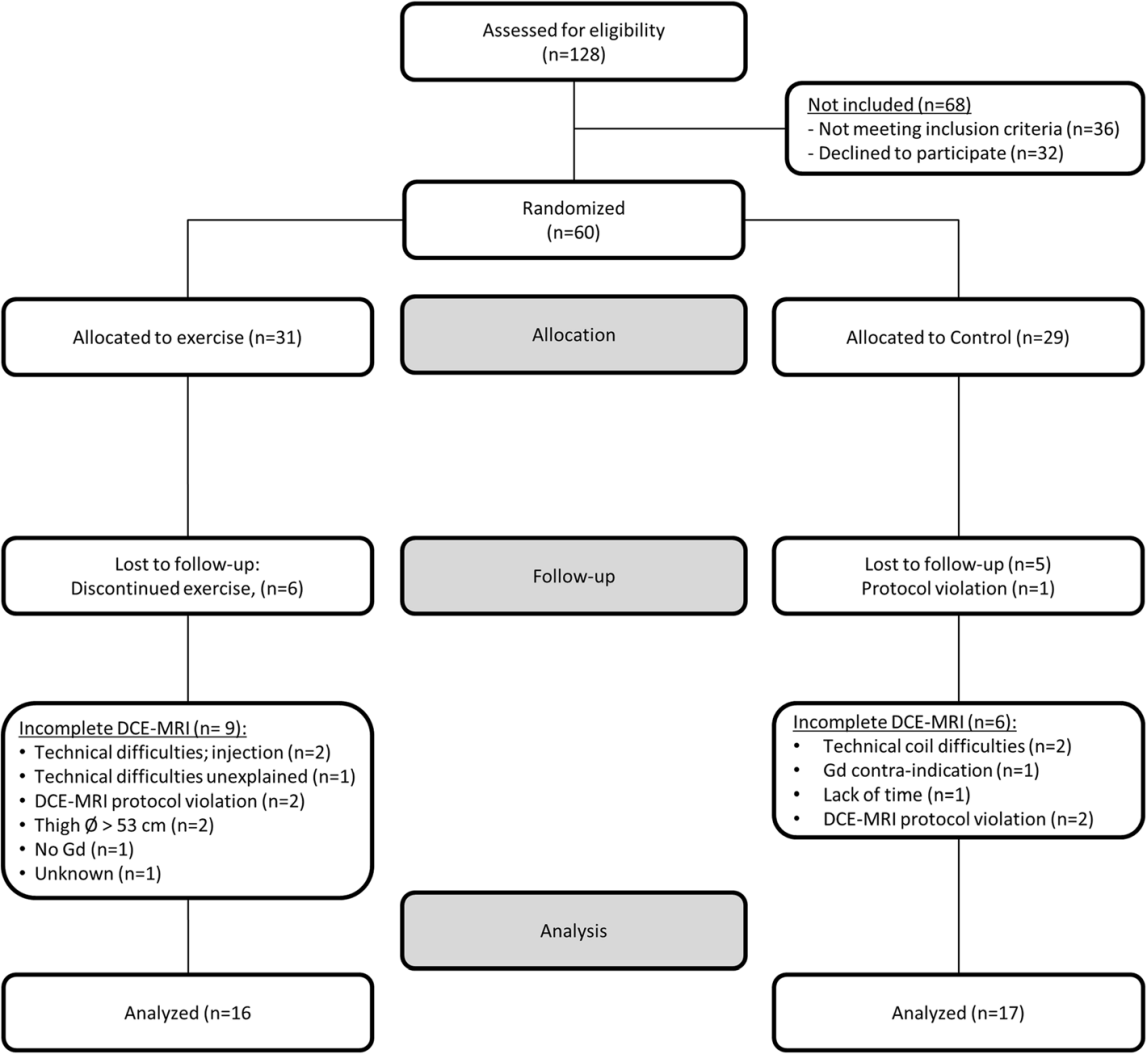
Flow chart

As pre-specified in the main trial protocol, the study focuses on individuals who adhere to the protocol. The per-protocol population was defined as participants with 12 or fewer incidences of non-attendance at exercise sessions in the ET group, and no exercise in the CG. The present exploratory analyses aim at investigating the effects of exercise on muscle perfusion in the peri-articular knee muscles; thus, only individuals included in the per-protocol population with complete DCE-MRI data sets at baseline and follow-up were included in the present analyses (Fig. 2).

Individual changes from baseline were calculated by subtracting the baseline value from the follow-up value for all outcomes. To assess group mean differences in the changes from baseline in all variables (purpose 1), analyses of covariance (ANCOVA) was used with change from baseline at follow-up as dependent variable, and baseline value of the dependent variable, treatment group, and gender (due to stratification by gender when randomising) as covariates [30]. For sensitivity purposes the analyses were repeated with further inclusion of age, weight and gender as covariates. All analyses were performed using SAS software (SAS Inc., Cary, NC, USA).

### Ancillary analyses

To explore whether changes from baseline to follow-up in the muscle perfusion variables were associated with changes in pain and function (KOOS) and physical performance measures (purpose 2), we assessed the rank correlation (Spearman’s rho) of changes from baseline between the DCE-MRI muscle perfusion variables, KOOS subscales, muscle strength, and 6MWT across the entire population.

Furthermore, we investigated the potential effect mediation of changes in the DCE-MRI perfusion variables for changes in KOOS pain in a post-hoc mediation analysis using a model-based approach with nonparametric bootstrap for variance estimation. The mediation models were adjusted for the same variables as the primary analysis (i.e. baseline KOOS pain and gender). We estimated an average causal mediation effect [31], i.e. the amount of the total effect that are mediated by a specific variable, as well as the proportion of the total effect that is mediated. The R software, version 3.5.1 (R Foundation for Statistical Computing, Vienna, Austria), with the R Package ‘mediation’ for Causal Mediation Analysis [32] was used.

## Results

Of the 60 included participants in the main trial, 31 were randomised to ET and 29 to the CG. The present analyses involved participants who adhered to the protocol with complete DCE-MRI data: 33 participants constituted the DCE-MRI per-protocol population (ET, *n* = 16; CG, *n* = 17). In the DCE-MRI per-protocol population group, imbalances were present in age, weight and BMI at baseline, with the ET group being older and less overweight. Details of study flow are given in Fig. 2. For baseline characteristics and data, see Table 2. There were no statistically significant differences between the DCE-MRI per-protocol population and the population not included in this study (ET, *n* = 15; CG, *n* = 12).

**Table 2:** Baseline characteristics of the patients: Demographics, self-reported symptoms (KOOS), DCE-MRI perfusion variables, and physical function

	Exercise therapy group		Control group	
	Randomised (*n* = 31)	Per-protocol (*n* = 16)	Randomised (*n* = 29)	Per-protocol (*n* = 17)
	Mean (SD)	Mean (SD)	Mean (SD)	Mean (SD)
Demographic Characteristics				
Age (years)	65.9 (8.5)	67.2 (8.2)	61.3 (7.1)	60.6 (7.2)
Female, no (%)	27 (87)	14 (87.5)	21 (71)	12 (70.6)
Height (m)	1.69 (0.08)	1.67 (0.07)	1.72 (0.09)	1.72 (0.11)
Weight (kg)	81.9 (14.1)	77.3 (8.3)	83.3 (15.0)	85.8 (14.2)
BMI (kg/m^2^)	28.7 (4.2)	27.7 (3.4)	28.1 (4.5)	29.0 (4.0)
KOOS				
Pain	56.5 (14.8)	52.8 (18.1)	63.3 (12.4)	61.3 (9.8)
Symptoms	58.3 (16.8)	57.4 (18.2)	65.5 (19.5)	63.9 (17.8)
Function in Daily Living	65.0 (14.0)	61.9 (17.3)	74.2 (13.9)	76.3 (10.9)
Knee-Related Quality of Life	27.6 (19.2)	37.9 (15.6)	34.7 (20.9)	44.1 (16.8)
Function in Sports and Recreation	37.1 (14.2)	25.0 (16.0)	44.2 (14.4)	37.4 (23.0)
DCE-MRI Perfusion variables				
Total Muscle VOI				
Nvoxel%	–	56.1 (15.7)	–	57.7(14.7)
IRExNvoxel	–	334.0 (171.5)	–	386.7 (230.2)
IRExNvoxel%	–	0.30 (0.15)	–	0.18 (0.07)
MExNvoxel	–	81,491.0 (26,054.0)	–	91,236.6 (29,556.8)
MExNvoxel%	–	72.8 (21.5)	–	73.4 (20.7)
Extensor VOI				
Nvoxel%	–	48.6 (16.0)	–	45.0 (19.5)
IRExNvoxel	–	98.1 (82.5)	–	113.0 (108.9)
IRExNvoxel%	–	0.39 (0.21)	–	0.4 (0.3)
MExNvoxel	–	16,553.4 (9817.4)	–	16,488.1 (10,599.5)
MExNvoxel%	–	67.1 (23.7)	–	60.6 (27.5)
Flexor VOI				
Nvoxel%	–	58.1 (16.6)	–	60.9 (13.8)
IRExNvoxel	–	236.1 (116.1)	–	273.7(150.0)
IRExNvoxel%	–	0.27 (0.15)	–	0.28 (0.16)
MExNvoxel	–	64,937.5 (17,987.4)	–	74,748.5 (21,639.4)
MExNvoxel%	–	74.3 (22.3)	–	76.5 (19.3)
Physical function				
Muscle strength Knee extension, Nm				
0°/s	–	103.1 (21.2)	–	124.1 (35.7)
60°/s	–	81.2 (24.8)	–	91.0 (29.9)
120°/s	–	69.3 (20.7)	–	86.9 (29.1)
180°/s	–	60.3 (17.0)	–	76.9 (26.8)
Muscle strength knee flexion, Nm				
0°/s	–	48.7 (17.3)	–	63.4 (22.8)
60°/s	–	38.1 (14.6)	–	49.0 (20.0)
120°/s	–	34.2 (13.8)	–	45.4 (19.7)
180°/s	–	32.4 (12.1)	–	44.1 (17.9)
6-min walk distance, m	–	494.4 (93.8)	–	541.2 (84.7)

*Abbreviations*: *SD* Standard deviation, *BMI* Body mass index, *KOOS* The Knee Injury and Osteoarthritis Outcome Score, *DCE-MRI* Dynamic contrast enhanced magnetic resonance imaging, *Total Muscle VOI* A volume of interest consisting of the summed and averaged peri-articular knee extensor and flexor muscle ROIs, *Extensor VOI* A volume of interest consisting of summed and averaged ROIs of the peri-articular knee extensor muscles, *Flexor VOI* A volume of interest consisting of summed and averaged ROIs of the peri-articular knee flexor muscles, *Nvoxel%* Proportion of Highly Perfused Voxels (%), *IRExNvoxel* Initial Rate of Enhancement Composite Score, *IRExNvoxel%* Initial Rate of Enhancement Index, *MExNvoxel* Maximal Enhancement Composite Score, *MExNvoxel%* Maximal Enhancement index

### DCE-MRI perfusion variables

At follow-up there were statistically significant group differences in changes from baseline in all muscle perfusion variables in favor of ET in the Total Muscle and Flexor VOIs showing a relative increase in the ET group compared with the CG.

Furthermore, in the Extensor VOI, the changes in Initial Rate of Enhancement Composite Score (IRExNvoxel) and Initial Rate of Enhancement Index (IRExNvoxel%) were statistically significantly different in favor of ET, showing similar patterns as for the Total Muscle and Flexor VOIs, with a relative increase in the ET group compared with the CG. The results are summarized in Table 3.

**Table 3:** Comparison of changes from baseline in outcomes adjusted for baseline values and gender

	Exercise group	Control group	Comparison	
	Mean (SE)	Mean (SE)	Mean Difference (95% CI)	*P*-value
KOOS changes from baseline				
Pain	8.5 (2.5)	−2.2 (2.5)	10.7 (3.3, 18.1)	0.006
Symptoms	3.7 (3.1)	−0.9 (3.0)	4.6 (−4.3, 13.6)	0.298
Function in Daily Living	6.9 (3.0)	0.1 (2.9)	6.7 (−2.3, 15.8)	0.140
Knee-Related Quality of Life	6.2 (3.7)	−2.2 (3.5)	8.4 (−2.3, 19.0)	0.118
Function in Sports and Recreation	9.2 (5.8)	−1.0 (5.6)	10.3 (−6.7, 27.2)	0.245
DCE-MRI perfusion variables changes from baseline				
Total Muscle VOI				
Nvoxel%	2.6 (2.3)	−5.2 (2.3)	7.7 (1.0, 14.5)	0.026
IRExNvoxel	− 51.6 (27.8)	− 154.6 (27.0)	103.0 (22.1, 183.9)	0.014
IRExNvoxel%	−0.03 (0.02)	−0.13 (0.02)	0.1 (0.03, 0.17)	0.008
MExNvoxel	1409.8 (3749.7)	− 10,032.9 (3632.9)	11,443.0 (− 531.4, 22,354.0)	0.041
MExNvoxel%	2.3 (3.1)	−8.3 (3.0)	10.6 (1.7, 19.4)	0.021
Extensor VOI				
Nvoxel%	0.3 (3.2)	−7.1 (3.1)	7.4 (−1.9, 16.6)	0.114
IRExNvoxel	−19.1(11.9)	−58.6 (11.6)	39.6 (5.1, 74.1)	0.026
IRExNvoxel%	−0.03 (0.04)	−0.21 (0.04)	0.18 (0.06, 0.3)	0.005
MExNvoxel	− 1093.6 (1364.8)	− 3532.2 (1323.2)	2438.6 (− 1.491.4, 6368.6)	0.215
MExNvoxel%	−0.4 (4.6)	−12.0 (4.5)	11.6 (− 1.8, 25.0)	0.087
Flexor VOI				
Nvoxel%	3.2 (2.2)	−5.2 (2.1)	8.5 (2.2, 14.8)	0.010
IRExNvoxel	−31.8 (18.6)	− 96.5 (18.0)	64.6 (10.4, 118.8)	0.021
IRExNvoxel%	−0.03 (0.02)	− 0.11(0.02)	0.08 (0.02, 0.14)	0.015
MExNvoxel	3247.0 (2861.0)	− 7200.6 (2770.1)	10,448 (2039.6, 18,856.0)	0.017
MExNvoxel%	3.2 (2.8)	−8.0 (2.7)	11.1 (3.0, 19.3)	0.009
Physical function changes from baseline				
Muscle strength Knee extension, Nm				
0°/s	−1.0 (4.3)	4.9 (4.1)	−5.8 (− 18.7, 7.0)	0.355
60°/s	−5.2 (3.0)	1.0 (2.9)	−6.2 (−15.0, 2.6)	0.161
120°/s	−5.3 (2.8)	−2.5 (2.7)	−2.8 (−11.1, 5.5)	0.488
180°/s	− 2.7 (2.8)	−1.3 (2.7)	−1.4 (−9.8, 7.0)	0.728
Muscle strength knee flexion, Nm				
0°/s	5.1 (3.0)	2.2 (2.9)	3.0 (−6.2, 12.1)	0.502
60°/s	1.2 (2.4)	2.5 (2.3)	−1.3 (−8.5, 5.9)	0.708
120°/s	−0.02 (1.8)	4.0 (1.8)	−4.0 (−9.4, 1.4)	0.146
180°/s	−0.6 (2.0)	2.3 (2.0)	−2.9 (−9.1, 3.2)	0.336
6-min walk distance, m	39.8 (13.7)	1.3 (12.8)	38.4 (−0.76, 77.6)	0.054

ANCOVA Analysis of covariance with each dependent variable adjusted for its baseline value and gender.

*Abbreviations*: *Mean* Least Squares means (covariate adjusted means), *SE* Standard Error, *CI* Confidence interval, *KOOS* The Knee Injury and Osteoarthritis Outcome Score, *DCE-MRI* Dynamic contrast enhanced magnetic resonance imaging, *Total Muscle VOI* A volume of interest consisting of the summed and averaged peri-articular knee extensor and flexor muscle ROIs, *Extensor VOI* A volume of interest consisting of summed and averaged ROIs of the peri-articular knee extensor muscles, *Flexor VOI* A volume of interest consisting of summed and averaged ROIs of the peri-articular knee flexor muscles, *Nvoxel%* Proportion of Highly Perfused Voxels (%), *IRExNvoxel* Initial Rate of Enhancement Composite Score, *IRExNvoxel%* Initial Rate of Enhancement Index, *MExNvoxel* Maximal Enhancement Composite Score, *MExNvoxel%* Maximal Enhancement index

### KOOS questionnaire

There was a statistically significant and clinically relevant group difference (10.7 KOOS points (95%CI 3.3 to 18.1)) in changes from baseline in KOOS pain in favor of ET similar to the per-protocol population in the main study [7]. No statistically significant group differences were observed in the other KOOS subscales (Table 3).

### Physical performance measures

No statistically significant group differences were observed in changes from baseline to follow-up in the muscle strength variables and in 6MWT (Table 3).

### Sensitivity analyses

The results were robust to sensitivity analysis, with only slight changes in the estimates (see the Additional file 1).

### Ancillary analyses

**Fig. 3:**
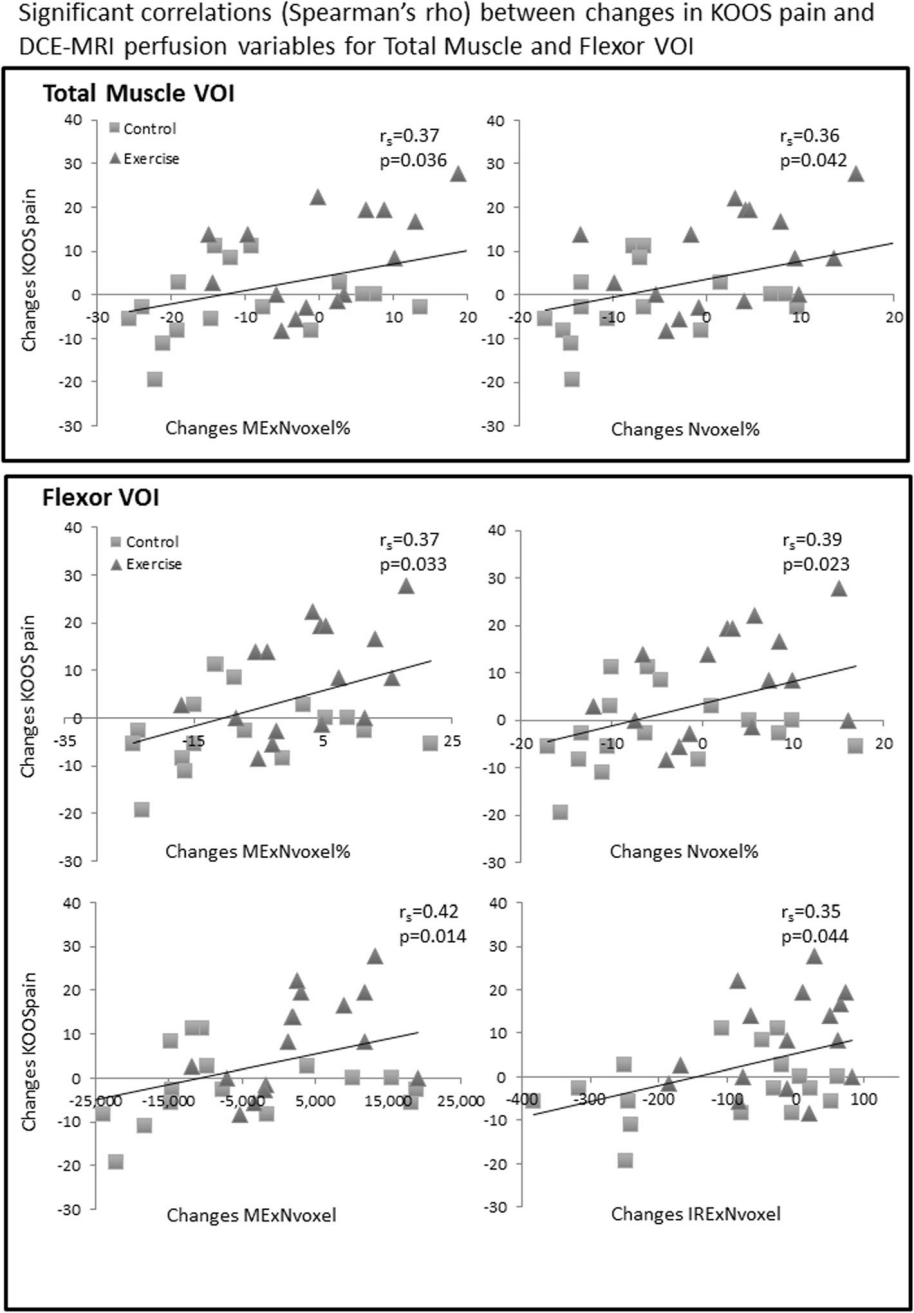
Scatterplots of significant correlations

Changes from baseline in KOOS pain were positively correlated with changes in The Proportion of Highly Perfused Voxels (%) (Nvoxel%) and Maximal Enhancement Index (MExNvoxel%) in the Total and Flexor VOIs (r ≥ 0.36, *P* ≤ 0.042). Further, changes in KOOS pain were positively correlated with changes in IRE Composite score (IRExNvoxel) and ME Composite score (MExNvoxel) in the Flexor VOI (r ≥ 0.35, *P* ≤ 0.044). This indicates that pain reduction was associated with increase in the degree and area of perfusion of the peri-articular muscles (Fig. 3).

No statistically significant associations were found between changes in muscle perfusion variables and the remaining 4 KOOS subscales (*r* < 0.18, *P* > 0.317). Changes from baseline in isometric knee extensor muscle strength were the only muscle strength variable which correlated significantly with changes in the muscle perfusion variables (the highest Spearman’s rho = − 0.46, *P* = 0.028). No other statistically significant associations were found between changes in muscle perfusion variables and changes in the variables of physical performance (highest Spearman’s rho = − 0.38, *P* = 0.076) (See the Additional file 1 for correlation matrix).

Over all, in the comparison between exercise and a no-attention control group, for changes in muscle perfusion variables (DCE-MRI) there was no statistically significant mediation of the pain-reducing effect of exercise in patients with knee OA, except for three muscle perfusion variables in the Flexor VOI. These were initial Rate of Enhancement Composite Score (IRExNvoxel), Maximal Enhancement Composite Score (MExNvoxel) and Maximal Enhancement index (MExNvoxel%) (Table 4), mediating 26% (95% CI, 0 to 1.24), 28% (95% CI, 1 to 1.71) and 28% (95% CI, 1 to 1.46), of the total effect of the pain-reducing effect of exercise, respectively. However, the upper limit of the 95% CI of the estimated proportions mediated suggest a potential mediation of effects on pain by all muscle perfusion variables (DCE-MRI) (Table 4).

**Table 4:** The mediation effect of changes in DCE-MRI perfusion variables for changes in KOOS pain

	Estimate (95%CIs) and *P*-value^a^	Proportion mediated (95% CI)^a^
Total effect	10.7 (3.3 to 18.1), *P* = 0.006	–
Mediator		
TotalMuscle		
Nvoxel%	2.1 (−0.1 to 9.6), *P* = 0.116	0.20 (−0.02 to 1.23)
IRExNvoxel	2.3 (−0.4 to 6.3), *P* = 0.092	0.21 (−0.06 to 0.81)
IRExNvoxel%	1.9 (−1.2 to 6.0), *P* = 0.230	0.18 (−0.15 to 0.88)
MExNvoxel	2.1 (−0.1 to 7.0), *P* = 0.094	0.19 (−0.02 to 0.88)
MExNvoxel%	2.1 (−0.1 to 9.5), *P* = 0.116	0.20 (−0.01 to 1.03)
Extensor		
Nvoxel%	0.6 (−0.7 to 4.3), *P* = 0.476	0.05 (−0.07 to 0.54)
IRExNvoxel	0.9 (−1.8 to 3.1), *P* = 0.468	0.09 (−0.26 to 0.35)
IRExNvoxel%	1.3 (−3.6 to 5.1), *P* = 0.496	0.12 (−0.43 to 0.53)
MExNvoxel	0.2 (−0.6 to 2.3), *P* = 0.630	0.02 (−0.07 to 0.30)
MExNvoxel%	0.5 (−0.7 to 3.9), *P* = 0.494	0.05 (−0.09 to 0.44)
Flexor		
Nvoxel%	2.8 (0.0 to 10.8), *P* = 0.060	0.26 (0.00 to 1.38)
IRExNvoxel	2.8 (0.0 to 7.7), *P* = 0.048	0.26 (0.00 to 1.24)
IRExNvoxel%	1.8 (−0.7 to 5.5), *P* = 0.182	0.17 (−0.08 to 0.82)
MExNvoxel	3.0 (0.1 to 12.3), *P* = 0.040	0.28 (0.01 to 1.71)
MExNvoxel%	3.0 (0.1 to 11.5), *P* = 0.038	0.28 (0.01 to 1.46)

^a^The mediation effect of changes in the DCE-MRI perfusion variables for changes in KOOS pain were calculated in a post-hoc mediation analysis using a model-based approach with nonparametric bootstrap for variance estimation. The models were adjusted for the same variables as the primary analysis (i.e. baseline KOOS pain and gender). An average causal mediation effect was estimated

*Abbreviations*: *CI* Confidence interval, *KOOS* The Knee Injury and Osteoarthritis Outcome Score, *DCE-MRI* Dynamic contrast enhanced magnetic resonance imaging, *Total Muscle VOI* A volume of interest consisting of the summed and averaged peri-articular knee extensor and flexor muscle ROIs, *Extensor VOI* A volume of interest consisting of summed and averaged ROIs of the peri-articular knee extensor muscles, *Flexor VOI* A volume of interest consisting of summed and averaged ROIs of the peri-articular knee flexor muscles, *Nvoxel%* Proportion of Highly Perfused Voxels (%), *IRExNvoxel* Initial Rate of Enhancement Composite Score, *IRExNvoxel%* Initial Rate of Enhancement Index, *MExNvoxel* Maximal Enhancement Composite Score, *MExNvoxel%* Maximal Enhancement index

## Discussion

Our results suggest that exercise therapy sustain peri-articular knee muscle perfusion compared to a no attention CG, as the participants in the ET exhibited a constant level of muscle perfusion in contrast to decreased perfusion in the CG over the 12-week intervention period. Besides the differences in muscle perfusion, exercise had a clinically relevant effect on self-reported pain. Further, across the population, changes in muscle perfusion were associated with changes in self-reported pain but not with physical function and performance. Although the mediation analyses overall were not statistically significant, all effects were in the expected direction. Altogether, these results indicate that muscle perfusion may be part of the underlying mechanisms of the pain-relieving effects of exercise. The present results extend our previous cross-sectional observation that more widespread muscle perfusion associates with less pain [12] and support the notion that muscle perfusion plays a role in the effects of exercise on knee OA pain [12].

### Mechanisms of changed muscle perfusion

The current study does not provide insight into the underlying mechanism of changed muscle perfusion. Exercise causes structural adaptations in the muscles, such as increased capillarisation or angiogenesis as a physiological response [9–11]. Based on the present results, it could be speculated that exercise in knee OA prevents such loss of capillarisation in the muscle tissue. Exercise programs specifically aiming at physiological muscle adaptations (e.g. resistance or cardiovascular training) may prove even more effective in inducing changes in muscle perfusion and are also beneficial in terms of pain [33, 34].

### Evaluating the effects of exercise

Pain experience is affected by psychological variables, such as depression and quality of life [35, 36], and exercise is likely to change these [37–39]. It is plausible that exercise-induced pain reductions may also be mediated through improvements in psychological well-being.

As exercise therapy cannot be blinded to the provider or to the patient, the observed clinical effects may be facilitated by effects associated with attention and study participation [8]. However, as the DCE-MRI assessment of muscle perfusion is a truly objective method, this study provides evidence of biological mechanisms associated with the pain-relieving effects of exercise therapy. Unlike radiographs, where there is discordance between radiographic findings and pain [40], and a poor short-term responsiveness to detecting change [41], muscle perfusion may be a potential objective marker of exercise-induced pain reduction (and potentially other treatments) on a structural level in knee OA.

Increased perfusion of intra-articular structures (e.g. synovium and the infra-patellar fat pad) are conventionally interpreted as signs of inflammation, and studies have repeatedly shown that intra-articular perfusion associates with a higher degree of pain [15–17, 27]. Our current and previous results [12] differ from these observations as the pain–perfusion relationship in the peri-articular muscle tissue is opposite. This could suggest a possible trade-off between intra-articular and peri-articular perfusion in the response to inflammation or treatment with subsequent changes in pain. This proposed mechanism needs further exploration.

We assessed knee OA pain using the KOOS questionnaire and found clinically relevant group differences that exceed the minimal clinically important difference (MCID) of 8–10 KOOS points as suggested by the developers of the questionnaire (www.koos.nu). This increases the relevance of our findings. In contrast, the MCIDs in the muscle perfusion variables have not been established, which encumbers interpretation of our results. However, the observed differences in muscle perfusion variables were all beyond the smallest detectable changes, and the reliability of the image analyses was satisfactory, which altogether strengthens our confidence in the results.

When evaluating the effects of exercise in knee OA, patient-reported outcomes (PROs) and performance-based tests are recommended and frequently used [5, 42, 43]. PROs and performance-based tests are affected by patient-provider interaction [44, 45], which is an important source of bias when the intervention cannot be blinded. Furthermore, the validity of performance-based tests (e.g. muscle strength) may be challenged by pain or fear of pain during the test [46, 47]. This means that a high degree of participant cooperation is necessary, which increases the risks of measurement bias and underlines the need for objective and sensitive measures in the evaluation of the underlying effects of exercise. DCE-MRI is such an objective method, and we applied it in the analysis of muscle perfusion, which is novel within OA research.

### Strengths and limitations

The use of a rigorous randomised study design with pre-specified methods using standardised and validated image analyses with high reproducibility are major strengths of our study. On the other hand, the limited sample size and the many variables and statistical tests performed increase the chances for multiplicity. The fact that the study was designed and powered for another outcome are limitations and calls for replication in larger studies specifically designed for DCE-MRI. Nevertheless, we could detect clinically meaningful important signals associated with the effects of exercise, a universally recommended treatment for knee OA. Another important aspect to consider is the per protocol design of the study. This was done to investigate the underlying mechanisms of exercise and thus it is necessary to focus on the participants who had complete DCE-MRI assessments at both baseline and follow-up and who had adhered to the protocol, i.e. received the allocated intervention (exercise vs control). This is fundamentally different from intention to treat study designs and therefore this study cannot be used to inform clinical practice. An important limitation is that out of 14 potential mediation analyses (corresponding to KOOS subdomains, muscle strength measurements, and 6-min walk distance) we only analysed mediation of effects on KOOS pain as this was the only outcome with significant group differences. This analysis did not support that changes in muscle perfusion mediate pain improvements.

## Conclusion

The pain-reducing effects of a 12-week exercise program are associated with changes in muscle perfusion quantified by DCE-MRI in individuals with knee OA, but whether the effects are mediated by muscle perfusion changes remains unclear.

## Supplementary information

**Additional file 1:** Supplementary information.

## Data Availability

The datasets analysed during the current study are available from the corresponding author on reasonable request.

## Abbreviations

6MWT: The six-minute walking test
ANCOVA: Analyses of covariance
CG: Control group
DCE-MRI: Dynamic contrast enhanced magnetic resonance imaging
ET: Exercise therapy
Gd: Gadolinium
KOOS: The knee Injury and Osteoarthritis Outcome Score
K^trans^: Contrast transfer coefficient from blood to extracellular space over time (min-1) and thus a measure of capillary permeability calculated using extended Tofts’s model.
MCID: Minimal clinically important difference
OA: Osteoarthritis
PROs: Patient-reported outcomes
ROIs: Regions of interest
TICs: Time-intensity-curves
VOIs: Volumes of interest
