# Supplementary material for: Exercise-induced pain changes associate with changes in muscle perfusion in knee osteoarthritis: exploratory outcome analyses of a randomised controlled trial
Source: BMC Musculoskelet Disord. 2019 Oct 27;20:491. doi: 10.1186/s12891-019-2858-8 (PMC6815355; doi:10.1186/s12891-019-2858-8)
Supplement: Supplementary file 1 — Additional file 1: Supplementary information. [file 12891_2019_2858_MOESM1_ESM.docx]

# Supplementary information for the article

Exercise-induced pain changes associate with changes in muscle perfusion in knee osteoarthritis: exploratory outcome analyses of a randomised controlled trial

**Authored by**

^1^Elisabeth Bandak, PT, MSc., ^1,2^Mikael Boesen, MD, Phd, ^1^Henning Bliddal, MD, DMSc,^1,2^Robert GC Riis, MD,PhD, , ^1,3^Sabrina Mai Nielsen, MSc, ^1^Louise Klokker, PT, PhD, ^1,4^Cecilie Bartholdy, PT, MSc, ^2^Janus Damm Nybing, MSc ^1,4^Marius Henriksen, PT, PhD

^1^The Parker Institute, Copenhagen University Hospital, Bispebjerg and Frederiksberg, Copenhagen, Denmark

^2^Department of Radiology, Copenhagen University Hospital, Bispebjerg and Frederiksberg, Copenhagen, Denmark

^3^Research Unit of Rheumatology, Department of Clinical Research, University of Southern Denmark, Odense University Hospital, Odense, Denmark

^4^ Department of physical and occupational therapy, Copenhagen University Hospital, Bispebjerg and Frederiksberg, Copenhagen, Denmark

**LIST OF CONTENT**

1. Detailed protocol for outcome measures
2. MRI-protocol
3. Table S1: Intra- and inter-observer reproducibility of the method
4. Table S2: Sensitivity analysis
5. Table S3: Ancillary analyses
6. Description of the exercise program
7. References

**1. DETAILED PROTOCOL FOR OUTCOME MEASURES**

**1.1 The Knee injury and Osteoarthritis Outcome Score (KOOS)**

To assess the participants’ pain, disease impact, and physical function the Knee injury and Osteoarthritis Outcome Score (KOOS)(1) questionnaire was applied. KOOS is a self-administered patient-reported outcome measure, assessing five domains of importance to patients with knee osteoarthritis (OA) (Pain, Symptoms, Function in Daily Living, Function in Sport and Recreation, and Knee Related Quality of Life) relating to target knee symptoms during the previous week. Each domain includes individual items answered on 0-4 Likert scale. A normalized 0-100 score (0 indicating the worst, 100 indicating the best) are calculated for all domains (1).

**1.2 Physical performance**

1.2.1 Muscle strength

Muscle strength of the target knee muscles was assessed for the quadriceps (extension) and hamstring muscles (flexion). The tests were performed isometrically (0°/sec) and during concentric isokinetic contractions at 60°/sec, 120°/sec, and 180°/sec using an isokinetic dynamometer (Biodex System4 Pro, Biodex Medical System, NY, USA). The dynamometer records the torque (Nm) produced by muscle contractions.

The isometric test was done at 60° knee flexion as this position gives close-to-optimum muscle lengths for both quadriceps and hamstrings in order to produce maximal force. The isokinetic tests were done in a range from 90° knee flexion to full extension.

At each angular velocity, the protocol comprised of 6 successive maximal efforts alternating between knee extension and knee flexion. Each contraction lasted 5 seconds with a 10 second pause between contractions.

1.2.2 Six-minutes walking distance

The Six-Minute Walk-Test (6MWT) is recommended to use as a measure of physical performance in knee OA (4-6). The test was performed in an undisturbed 50-meter corridor. Subjects were instructed to walk as fast as they could. They were allowed to stop or rest during the test if necessary, and walking aids were allowed. The distance in meters covered during the 6 minutes was the outcome of the test.

**1.3 Dynamic contrast enhanced magnetic resonance imaging (DCE-MRI)**

As previously described in details (7;8), DCE-MRI analysis is based on changes in signal intensity over time for each voxel within the volume of interest (VOI). The signal intensity changes are calculated relatively to the baseline signal intensity and the changes over time can be plotted as time-intensity-curves (TICs). Tissues with high perfusion (such as an artery) has a characteristic rapid increase in signal intensity, which reaches a plateau and a subsequent rapid decrease often referred to as washout (Figure 1A). Tissue with lower perfusion shows a slower increase and oftentimes does not reach a plateau or washout phase.

The dedicated software uses a robust classification scheme(7) that based on the shape of linear approximations of the TICs, assigned each voxel to one of the following enhancement patterns with an associated color code for visualization: “No enhancement” (voxels with no contrast uptake; no color), “Persistent” (voxels with uptake but no plateau phase; blue), “Plateau” (voxels that reach plateau but without washout phase; green), and “Washout” (voxels that reach a washout phase; red)(7;9;10)(Figure 1B).

The following 3 perfusion variables were calculated for each VOI: 1) Number of Highly Perfused Voxels, defined as the number of voxels within a VOI classified as having a “Plateau” or “Washout” enhancement patterns, thus reflecting the number of highest perfused voxels and thereby a measure of the most perfused tissue (7;11;12). 2) Maximal enhancement (ME), the highest mean signal intensity value relative to the baseline signal intensity; 3) Initial rate of enhancement (IRE) i.e. the upslope on the TIC measured as the mean relative increase in signal intensity per second (%/s) IRE and ME were quantified as average measures of the whole segmented region.

As outcomes in the present study and according to previous use of DCE-MRI in the quantification of tissue perfusion(8;11;13-15), we present the following 5 DCE-MRI outcomes (Table 1) that reflect the degree and extent of perfusion in the peri-articular muscle tissue: 1) Proportion of Highly Perfused Voxels defined as the Number of Highly Perfused Voxels (defined above) in relation to the total number of voxels in the VOI; 2) Maximal Enhancement Composite Score calculated as the product of ME and the Number of Highly Perfused Voxels and 3) Maximal Enhancement Index calculated as the product of ME and the Proportion of Highly Perfused Voxels; 4) Initial Rate of Enhancement Composite Score calculated as the product of IRE and Number of Highly Perfused Voxels and 5) Initial Rate of Enhancement Index calculated as the product of IRE and Proportion of Highly Perfused Voxels (11).

**2.0 MRI protocol**

The following MRI protocol was used: Gradient echo (GRE) scout (Slice thickness (ST) 8 mm, Field of View (FOV) 330 mm, Echo time (TE) 3.69 ms, Repetition time (TR) 7.7 ms, scan time 18 sec ; coronal and sagittal short tau inversion recovery (STIR) (ST 3 mm, FOV 160 mm, matrix 256x230, in plane resolution 0.7x0.6 mm, Inversion time (TI) 220 ms, TE 34 ms, TR 4350 ms, scan time 1min 48sec); coronal T1-weighted (T1w) turbo spin echo (TSE) (ST 3.5 mm, FOV 140 mm, matrix 384x288, in plane resolution 0.6x0.4 mm, TE 17 ms, TR 790 ms, scan time 2 min 24 sec); sagittal 3D proton density weighted (PDw) fat suppressed (FS) TSE SPACE (ST 0.6 mm, FOV 160mm, matrix 320x260mm, in plane resolution 0.6x0.6 mm, TE 44 ms, TR 1000 ms, scan time 9 min 26 sec);

In addition to the standard clinical MRI protocol a sagittal DCE T1 VIBE sequence was performed with a using 5 mm slices, temporal resolution of 9 sec and 30 repetitions using the following parameters: TE 1.9, TR 5.44, FA 15 degrees, FOV 160mm, matrix 192x138, in plane resolution1.2x0.8 mm, scan time 4 min 52 sec. During the third repetition an intravenous injection of 0.1 ml/kg body weight Gadolinium (Gd) contrast agent (Prohance®, Bracco Diagnostics Inc., Italy) using a power injector (2 ml/s) was administered. Due to the potential side effects, only participants with an estimated glomerular filtration rate (eGRF) > 60 ml/min/1.73m2 were administered Gd.

The DCE sequence was followed by a post contrast sagittal GRE 3D T1w VIBE (ST 0.6 mm, FOV 160 mm, matrix 192x138, in plane resolution 0.6x0.6 mm, Flip angle (FA) 10 degrees, TE 5.39 ms, TR 11.6 ms, scan time 3 min 19 seconds). The total imaging time was 25 minutes.

|  | Intra-observer reliability |  |  |  |  |  |  | Inter-observer reliability |  |  |  |  |  |  |
| --- | --- | --- | --- | --- | --- | --- | --- | --- | --- | --- | --- | --- | --- | --- |
| Total Muscle VOI | Intra class correlation coefficients  (95%CI) | Measurement errors (ME) | Mean  (SD) | Mean difference  (SD) | Smallest  Detectable Change (SDC) | Limits of Agreement (LOA) | LOA% | Intra class correlation coefficients  (95%CI) | Measurement errors (ME) | Mean  (SD) | Mean difference (SD) | Smallest Detectable Change (SDC) | Limits of Agreement (LOA) | LOA  % |
| Volume (voxels) | 0.99 (0.98;0.99) | 1,983.1 | 125,890.6  (27,968.2) | -650.2  (2804.5) | 5,496.9 | -6,147.1; 4,846.7 | 4.4 | 0.81 (-0.05;0.97) | 4,836.10 | 117,186.7  (27,901.4) | 18,058.0  (6548.4) | 12,834.8 | 5,223.2;  30,892.9 | 11.0 |
| Volume (cm^3^) | 0.99 (0.99;1.00) | 6.90 | 437.1 (97.1) | -2.26 (9.74) | 19.1 | -21.3; 16.8 | 4.4 | 0.81 (-0.05;0.97) | 1.15 | 406.9 (96.9) | 62.70 (22.7) | 44.6 | 18.14; 107.3 | 11.0 |
| Nvoxel% | 0.99 (0.98;1.00) | 0.71 | 48.0 (48.8) | 0.471 (1.003) | 2.0 | -1.5; 2.4 | 4.1 | 0.96 (0.04;0.99) | 1.15 | 50.1  (15.6) | -4.74  (1.7) | 3.3 | -8.0; -1.5 | 6.6 |
| IRExNvoxel | 1.00 (0.97;1.00) | 2.40 | 252.3 (241.7) | 5.32 (3.40) | 6.7 | -1.33; 11.98 | 2.6 | 0.99 (0.96;1.0) | 17.91 | 254.0 (190.2) | -8.7 (24.3) | 47.6 | -56.3; 38.9 | 18.8 |
| IRExNvoxel% | 1.00 (0.99;1.0) | 0.0041 | 0.196 (0.136) | 0.004 (0.006) | 0.01 | -0.007; 0.016; | 5.9 | 0.97 (0.13;1.0) | 0.012 | 0.213  (0.143) | -0.038 (0.016) | 0.03 | -0.07; -0.006 | 14.7 |
| MExNvoxel | 1.00 (0.99;1.00) | 587.0 | 75,798.3  (77,490.2) | 292.74 (830.14) | 1,627.1 | -1,334.3;  1,919.8 | 2.2 | 0.98 (0.70;1.00) | 2488.69 | 73,548.5  (29,895.1) | 4,207.0  (3,604.8) | 7065.5 | -2,858.5;  11,272.4 | 9.6 |
| MExNvoxel% | 1.00 (0.91;1.00) | 0.94 | 59.9 (60.6) | 0.65 (1.33) | 2.6 | -2.0; 3.3 | 4.4 | 0.96 (0.04;0.99) | 1.41 | 62.5 (19.9) | -5.9 (2.1) | 4.1 | -10.0; -1.8 | 6.5 |
| K^trans^ | **0.77 (0.3;0.94)** | **0.0043** | **0.017 (0.010)** | **0.004 (0.006)** | **0.01** | **0.016; -0.008** | **69.7** | **0.35 (-0.40;0.79)** | **0.009** | **0.017 (0.008)** | **-0.004 (0.009)** | **0.02** | **0.013; -0.021** | **96.3** |
| Intra- and inter-observer reproducibility of the method was tested for the Total VOI on a random, repeated subsample (n=10) with minimum 4 weeks between segmentations.  Intra class correlation coefficients were calculated as a reliability parameter (ICC) (3.1) using single measures and absolute agreement definition.  As agreement parameters, measurement errors (ME) were calculated as the square root of the residual mean square values obtained from analyses of variance, smallest detectable changes (SDC), and Limits of Agreement (LOA) (Mean diff+/-1.96SD). Further Limits of agreement % (LOA %): ((1.96xSD)/Mean difference) x 100%) was calculated.  Abbrevations*: SD*: standard deviation; *Total Muscle VOI*: a volume of interest consisting of the summed and averaged peri-articular knee extensor and flexor muscle ROIs. *Nvoxel%*: Proportion of Highly Perfused Voxels (%); *IRExNvoxel*: Initial Rate of Enhancement Composite Score; *IRExNvoxel%*: Initial Rate of Enhancement Index; *MExNvoxel*: Maximal Enhancement Composite Score; *MExNvoxel%*: Maximal Enhancement index; K^trans^: the volume transfer coefficient for passage of Gadolinium from blood vessels to the extraqcellurlar space over time (min^-1^). | | | | | | | | | | | | | | |

**3. Table S1**: Intra- and inter-observer reproducibility of the method

**4. Table S2**: Sensitivity analyses with each dependent variable adjusted for its baseline value, age, weight and gender.

|  | **Exercise group** | **Control group** | **Comparison** | | |
| --- | --- | --- | --- | --- | --- |
|  | Mean (SE) | Mean (SE) | Mean Difference (95% CI) | P value | |
| **KOOS** | | | | | |
| Pain | 10.6 (3.1) | -1.1 (2.9) | -11.7 (-20.07, -3.41) | | 0.008 |
| Symptoms | 6.00 (3.9) | 0.2 (3.5) | -5.8 (-15.82, 4.25) | | 0.247 |
| Function in Daily Living | 7.8 (3.9) | -0.1 (3.3) | -8.0 (-18.2, 2.5) | | 0.129 |
| Knee-Related Quality of Life | 9.0 (4.8) | -1.1 (4.1) | -10.2 (-22.0, 2.1) | | 0.100 |
| Function in Sports and Recreation | 12.4 (7.5) | -0.7 (6.8) | -13.1 (-32.9, 6.7) | | 0.185 |
| **DCE-MRI perfusion variables changes from baseline** | | | | | |
| Total Muscle VOI | | | | | |
| Nvoxel% | 4.1 (3.1) | -2.0 (2.6) | -6.1 (-14.0, 1.8) | | 0.125 |
| IRExNvoxel | -38.1 (35.9) | 164.0 (31.1) | -125.9 (-217.1, -34.7) | | 0.009 |
| IRExNvoxel% | -0.03 (0.03) | -0.14 (0.03) | -0.12 (-0.20, -0.03) | | 0.008 |
| MExNvoxel | 6,038.4 (4,644.1) | -4,497.9 (4,087.1) | -10,536.0 (-22,423.0, 1,350.0) | | 0.08 |
| MExNvoxel% | 3.9 (4.1) | -5.4 (3.5) | -9.3 (-19.9, 1.3) | | 0.084 |
| Extensor VOI | | | | | |
| Nvoxel% | 2.1 (4.2) | -6.1 (3.7) | -8.2 (-18.8, 2.5) | | 0.126 |
| IRExNvoxel | -10.8 (14.8) | -62.3 (13.0) | -51.5 (-89.1, -13.9) | | 0.009 |
| IRExNvoxel% | -0.01 (0.05) | -0.22 (0.05) | -0.21 (-0.35, -0.08) | | 0.003 |
| MExNvoxel | -286.3 (1737.4) | -3,449.8 (1,521.8) | -3,163.5 (-7,585.9, 1,258.9) | | 0.154 |
| MExNvoxel% | 2.0 (6.0) | -11.3 (5.3) | -13.3 (-28.7, 2.0) | | 0.086 |
| Flexor VOI | | | | | |
| Nvoxel% | 5.5 (2.9) | -1.57 (2.46) | -7.02 (-14.54, 0.50) | | 0.066 |
| IRExNvoxel | -24.5 (25.0) | -102.1 (21.1) | -77.6 (-140.8, -14.4) | | 0.018 |
| IRExNvoxel% | -0.03 (0.03) | -0.12 (0.02) | -0.09 (-0.17, -0.02) | | 0.018 |
| MExNvoxel | 7,636.7 (3,597.5) | -1,695.7 (3,136.0) | -9,332.4 (-18,620.0, -44.6) | | 0.049 |
| MExNvoxel% | 5.8 (3.9) | -4.5 (3.3) | 10.4 (-20.3, -0.4) | | 0.042 |
| **Physical function changes from baseline** | | | | | |
| Muscle strength Knee extension, Nm | | | | | |
| 0°/s | 7.4 (5.0) | 2.3 (3.8) | -5.1 (-18.2, 8.0) | | 0.422 |
| 60°/s | -3.2 (3.9) | -1.2 (3.2) | 2.0 (-7.7, 11.7) | | 0.673 |
| 120°/s | -8.2 (3.7) | -7.5 (3.1) | 0.7 (-8.3, 9.8) | | 0.868 |
| 180°/s | -2.7 (3.8) | -3.2 (3.2) | -0.4 (-9.8, 8.9) | | 0.923 |
| Muscle strength knee flexion, Nm | | | | | |
| 0°/s | 9.5 (4.4) | 2.6 (3.3) | -6.9 (-17.9, 4.2) | | 0.208 |
| 60°/s | 0.8 (3.2) | -0.08 (2.7) | -0.9 (-8.8, 7.1) | | 0.826 |
| 120°/s | -0.08 (2.35) | 1.65 (2.0) | 1.7 (-4.1, 7.6) | | 0.546 |
| 180°/s | 0.9 (2.8) | 3.0 (2.4) | 2.2 (-4.8, 9.1) | | 0.528 |
| 6-min walk distance, m | 47.1 (16.5) | 11.9 (14.7) | -35.2 (-76.6, 6.2) | | 0.092 |
| ANCOVA, Analysis of covariance with each dependent variable adjusted for its baseline value, age, weight and gender.  Abbreviations: *Mean*: Least Squared means; *SE*: Standard Error; *KOOS*: the Knee Injury and Osteoarthritis Outcome Score; *DCE-MRI*: dynamic contrast enhanced magnetic resonance imaging; *Total Muscle VOI*: a volume of interest consisting of the summed and averaged peri-articular knee extensor and flexor muscle ROIs. *Extensor VOI*: a volume of interest consisting of summed and averaged ROIs of the peri-articular knee extensor muscles. *Flexor VOI*: a volume of interest consisting of summed and averaged ROIs of the peri-articular knee flexor muscles; *Nvoxel%*: Proportion of Highly Perfused Voxels (%); *IRExNvoxel*: Initial Rate of Enhancement Composite Score; *IRExNvoxel%*: Initial Rate of Enhancement Index; *MExNvoxel*: Maximal Enhancement Composite Score; *MExNvoxel%*: Maximal Enhancement index. | | | | | |

**5. Table S3**: Ancillary analyses.

|  | **Total Muscle VOI** | | | | | **Flexor VOI** | | | | | | | **Extensor VOI** | | | | | | | | | |
| --- | --- | --- | --- | --- | --- | --- | --- | --- | --- | --- | --- | --- | --- | --- | --- | --- | --- | --- | --- | --- | --- | --- |
| **Changes in** | **Nvoxel%** | **IRExNvoxel** | **IRExNvoxel%** | **MExNvoxel** | **MExNvoxel%** | **Nvoxel%** | **IRExNvoxel** | **IRExNvoxel%** | **MExNvoxel** | | **MExNvoxel%** | | **Nvoxel%** | | **IRExNvoxel** | | **IRExNvoxel%** | | **MExNvoxel** | | **MExNvoxel%** | |
| **KOOS ADL** | 0.12 | 0.18 | 0.18 | 0.11 | 0.15 | 0.13 | 0.18 | 0.15 | 0.18 | | 0.13 | | 0.09 | | 0.08 | | 0.08 | | 0.02 | | 0.10 | |
|  | (0.514) | (0.311) | (0.321) | (0.558) | (0.396) | (0.473) | (0.305) | (0.401) | (0.317) | | (0.456 | | (0.609) | | (0.677) | | (0.656) | | (0.915) | | (0.574) | |
| **KOOS Pain** | 0.36 | 0.34 | 0.29 | 0.33 | 0.37 | 0.39 | 0.35 | 0.29 | 0.42 | | 0.37 | | 0.24 | | 0.22 | | 0.22 | | 0.13 | | 0.20 | |
|  | (0.042)* | (0.057) | (0.101) | (0.062) | (0.036)* | (0.023)* | (0.044)* | (0.097) | (0.014)* | | (0.033)* | | (0.180) | | (0.216) | | (0.208) | | (0.465) | | (0.266) | |
| **KOOS QOL** | 0.08 | 0.15 | 0.15 | 0.03 | 0.08 | 0.10 | 0.17 | 0.15 | 0.11 | | 0.06 | | 0.08 | | 0.04 | | 0.05 | | -0.01 | | 0.06 | |
|  | (0.655) | (0.417) | (0.393) | (0.856) | (0.664) | (0.576) | (0.350) | (0.396) | (0.557) | | (0.722) | | (0.670) | | (0.842) | | (0.780) | | (0.935) | | (0.736) | |
| **KOOS Sport/Req** | 0.17 | 0.09 | 0.12 | 0.05 | 0.16 | 0.14 | 0.11 | 0.10 | 0.10 | | 0.09 | | 0.08 | | -0.02 | | 0.02 | | -0.17 | | 0.07 | |
|  | (0.335) | (0.608) | (0.494) | (0.788) | (0.377) | (0.453) | (0.540) | (0.5)86 | (0.572) | | (0.627) | | (0.671) | | (0.925) | | (0.919) | | (0.357) | | (0.703) | |
| **KOOS Symp** | 0.09 | 0.17 | 0.16 | 0.09 | 0.09 | 0.07 | 0.17 | 0.14 | 0.11 | | 0.08 | | 0.02 | | 0.12 | | 0.17 | | 0.00 | | 0.01 | |
|  | (0.620) | (0.333) | (0.365) | (0.620) | (0.629) | (0.683) | (0.3)36 | (0.444) | (0.534) | | (0.674) | | (0.918) | | (0.504) | | (0.332) | | (0.994) | | (0.972) | |
| **6-min walk distance. m** | 0.12 | -0.02 | 0.01 | 0.13 | 0.13 | 0.20 | 0.08 | 0.09 | 0.19 | | 0.23 | | -0.07 | | -0.16 | | -0.07 | | -0.05 | | -0.08 | |
|  | (0.502) | (0.905) | (0.940) | (0.490) | (0.484) | (0.278) | (0.646) | (0.629) | (0.303) | | (0.213) | | (0.715) | | (0.383) | | (0.711) | | (0.796) | | (0.681) | |
| **Muscle strength** |  | | | | |  | | | | | | |  | | | | | | | | | |
| **Extensor 0°/s** | -0.43 | -0.46 | -0.45 | -0.38 | -0.43 | -0.34 | -0.44 | -0.42 | -0.35 | | -0.38 | | -0.29 | | -0.32 | | -0.43 | | -0.14 | | -0.35 | |
|  | (0.043)* | (0.028)* | (0.029)* | (0.076) | (0.039)* | (0.108) | (0.035)* | (0.047)* | (0.100) | | (0.077) | | (0.176) | | (0.136) | | (0.042)* | | (0.532) | | (0.105) | |
| **Extensor 60°/s** | -0.15 | -0.07 | -0.09 | -0.15 | -0.11 | -0.06 | -0.02 | 0.01 | -0.12 | | -0.07 | | -0.17 | | -0.10 | | -0.17 | | -0.01 | | -0.16 | |
|  | (0.429) | (0.713) | (0.626) | (0.447) | (0.576) | (0.768) | (0.905) | (0.966) | (0.525) | | (0.717) | | (0.381) | | (0.618) | | (0.378) | | (0.977) | | (0.394) | |
| **Extensor 120°/s** | -0.35 | -0.07 | -0.10 | -0.27 | -0.28 | -0.29 | -0.08 | -0.03 | -0.34 | | -0.25 | | -0.31 | | 0.08 | | -0.18 | | 0.16 | | -0.30 | |
|  | (0.061) | (0.736) | (0.611) | (0.150) | (0.143) | (0.133) | (0.676) | (0.881) | (0.069) | | (0.194) | | (0.105) | | (0.681) | | (0.357) | | (0.422) | | (0.108) | |
| **Extensor 180°/s** | -0.07 | -0.06 | -0.14 | -0.02 | -0.10 | -0.03 | -0.03 | -0.02 | 0.01 | | -0.04 | | -0.06 | | -0.03 | | -0.21 | | 0.11 | | -0.11 | |
|  | (0.703) | (0.763) | (0.458) | (0.923) | (0.608) | (0.869) | (0.879) | (0.901) | (0.972) | | (0.843) | | (0.763) | | (0.897) | | (0.265) | | (0.557) | | (0.576) | |
| **Flexor 0°/s** | -0.03 | -0.21 | -0.16 | -0.02 | 0.02 | 0.05 | -0.23 | -0.17 | -0.03 | | 0.07 | | -0.01 | | -0.21 | | -0.30 | | -0.05 | | -0.04 | |
|  | (0.883) | (0.342) | (0.474) | (0.925) | (0.942) | (0.830) | (0.291) | (0.446) | (0.897) | | (0.764) | | (0.964) | | (0.337) | | (0.161) | | (0.812) | | (0.851) | |
| **Flexor 60°/s** | -0.15 | -0.18 | -0.19 | -0.16 | -0.10 | -0.03 | -0.18 | -0.16 | -0.10 | | -0.03 | | -0.19 | | -0.26 | | -0.20 | | -0.18 | | -0.14 | |
|  | (0.423) | (0.362) | (0.330) | (0.395) | (0.593) | (0.867) | (0.349) | (0.421) | (0.593) | | (0.881) | | (0.330) | | (0.174) | | (0.295) | | (0.352) | | (0.464) | |
| **Flexor 120°/s** | -0.15 | -0.04 | -0.05 | -0.19 | -0.10 | -0.08 | -0.08 | -0.03 | -0.19 | | -0.07 | | -0.21 | | -0.01 | | -0.15 | | 0.02 | | -0.18 | |
|  | (0.452) | (0.833) | (0.788) | (0.335) | (0.613) | (0.698) | (0.688) | (0.887) | (0.327) | | (0.711) | | (0.275) | | (0.974) | | (0.423) | | (0.905) | | (0.363) | |
| **Flexor 180°/s** | 0.22 | -0.20 | -0.20 | 0.13 | 0.17 | 0.33 | -0.13 | -0.11 | 0.20 | | 0.26 | | -0.07 | | -0.22 | | -0.31 | | -0.11 | | -0.11 | |
|  | (0.262) | (0.307) | (0.307) | (0.513) | (0.377) | (0.076) | (0.488) | (0.579) | (0.295) | (0.175) | | (0.738) | | (0.253) | | (0.106) | | (0.564) | | (0.579) | |  |
| Spearman Correlation Coefficients of changes, rho and (P-values) *:P≤0.05. Abbreviations: *KOOS:* Knee Injury and Osteoarthritis Outcome Score; *ADL:* Function in daily living; *QOL*: Knee related quality of life; *Sport/Rec:* Function in sport and recreation; *Total Muscle VOI*: a volume of interest consisting of the summed and averaged peri-articular knee extensor and flexor muscle ROIs. *Extensor VOI*: a volume of interest consisting of summed and averaged ROIs of the peri-articular knee extensor muscles. *Flexor VOI*: a volume of interest consisting of summed and averaged ROIs of the peri-articular knee flexor muscles; *Nvoxel%*: Proportion of Highly Perfused Voxels (%); *IRExNvoxel*: Initial Rate of Enhancement Composite Score; *IRExNvoxel%*: Initial Rate of Enhancement Index; *MExNvoxel*: Maximal Enhancement Composite Score; *MExNvoxel%*: Maximal Enhancement index. | | | | | | | | | | | | | | | | | | | | | |  |

**6.** Description of the exercise program

Individualized therapeutic exercise program for participants with
knee osteoarthritis

Developed by

Cecilie Bartholdy PT, Louise Klokker PT, MSc, and Marius Henriksen PT, MSc, PhD

This exercise program runs for 12 weeks, with exercise sessions 3 times per week. Each training session is scheduled to last approximately 60 minutes. The exercise is facility based and takes place in groups, under the supervision of one or more physical therapist. The program consists of a warm up and a circuit training program. The circuited program contains: Core stability training, hip stability training, hip muscle strengthening, and specific knee training focussing on coordination, stability, strengthening, and translational and functional tasks.

Focus of the exercises is on the quality of the performance – not quantity. Each exercise has a specific focus as specified below. During the initial sessions, emphasis is on the specified focus area and each participant is instructed and informed about the importance of doing every exercise correctly and with the proper technique. All exercises have several levels of difficulty. The physical therapist supervises each participant individually and adjusts the exercises and progression in difficulty of these, individually during the entire course of the program.

**Monitoring of knee pain**

A 0-10 numeric rating scale (0= ‘No pain’; 10=’Worst imaginable pain’; Figure 1) is used to monitor each participant’s current knee pain intensity before, during and after each training session. If a participant experiences knee pain of an intensity of 5 or more before or during a training session, the participant will be referred to the ‘rescue exercises program’ (see below) that reduces the physical demands to the knee. The pain ratings are recorded in the participant’s exercise diary at each session.

If unacceptable pain ratings (>5) persists more than 3 consecutive exercise sessions, the participant is referred to a rheumatologist associated with the program.


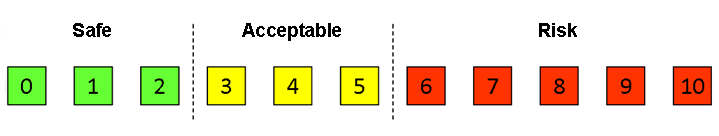


**Figure 1**: Visual numeric rating scale for pain monitoring ranging from 0 (no pain) to 10 (worst imaginable pain) with safe, acceptable, and risk pain zones indicated.

**1. Warm-up**

The warm-up period consists of 10 minutes ergometer cycling at 80-100 rpm or ‘morderate intensity (corresponding to 11-15 on Borg Rating of Perceived Exertion Scale; “somewhat hard”). During each warm-up period the rpm and Borg scale rating is noted in the exercise diary to ensure that the participant warms up at the same intensity or higher at each training session.

**2. Circuit Program**

The circuit program features 8 exercise stations, each with a main area in focus: Core stability, Hip stability, Hip muscle strengthening, Knee coordination/stability, Knee muscle strengthening, and Translation and Functional exercises. Every exercise is adjusted individually accordingly to each individual’s level and progress.

**2.1 Core stability**

**Purpose**: To maintain core stability and adequate trunk muscle activation control during limb movement.

**Focus**: Main focus is on keeping the lower back straight during limb movement. If the lower back cannot be held steady the participant should be instructed to take a short break and start again. From level D-F maintaining the body in frontal plan and making sure the pelvis does not drop is in focus.

**Time**: Level **A**-**D** the position is kept 2×1 minute.

**Repetitions**: Level **E**-**F**, 2×6-8 movement repetitions – left and right side.

**Progression:** The exercise is progressed from level **A (easiest) to F (most difficult)**.


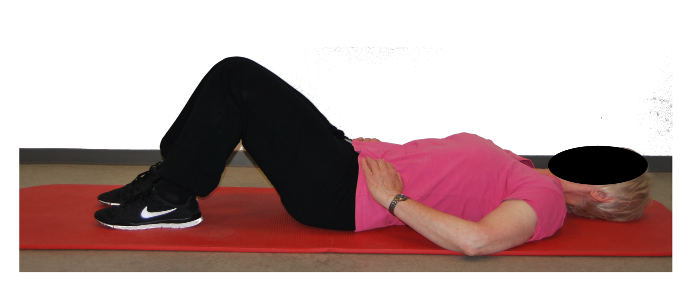
**A.** Selective abdominal activation exercise: Supine with feet placed on the ground. A finger is placed just medial to each anterior superior iliac spine to monitor selective activation of m. transversus abdominis without activation of the other abdominal muscles. Once this is possible, one hip is slowly externally rotated (lowering of the knee sideways towards the floor) and moved slowly back again, without loosing the tension in m. transversus abdominis (felt by the fingers), pelvic rotation, excessive activation of the other abdominal muscles, or forced breathing. The exercise is done with each leg, one at a time, until the correct muscle tension is sustained with good control during the hip movement.

**Progression**: When the participant is able to selectively activate m. transversus abdominis with no pelvic rotation, without holding their breath, or activating additional abdominal muscles, and perform the movement freely and effortless, the participant is progressed to next level.


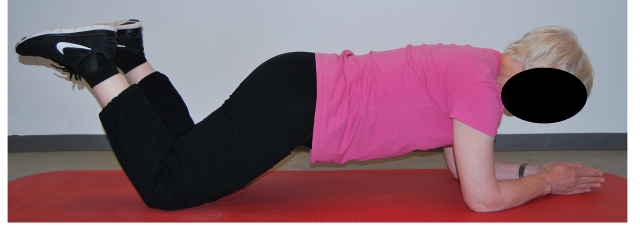
**B.** Kneeling Plank: Prone kneeling position resting on knees and elbows. The body is raised to form a straight line from the neck to the knees (a plank).

**Progression:** When the participant is able to perform the exercise without distinct exertion and good trunk control, progress to next level.


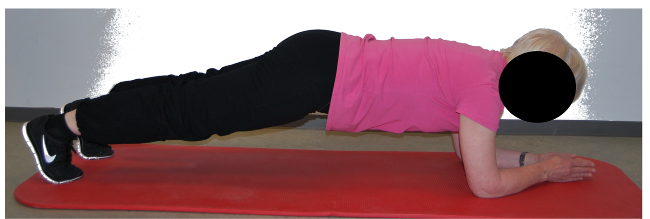
**C.** Full Plank: Forward lying with both elbows and

feet on the floor. The entire body is now lifted so the body weight is supported only by the elbows and toe tips. From head to toe the body is forming a straight line (a plank).

**Progression:** When the participant is able to perform the exercise without distinct exertion and show good control of the trunk, the participant is progressed to next level.


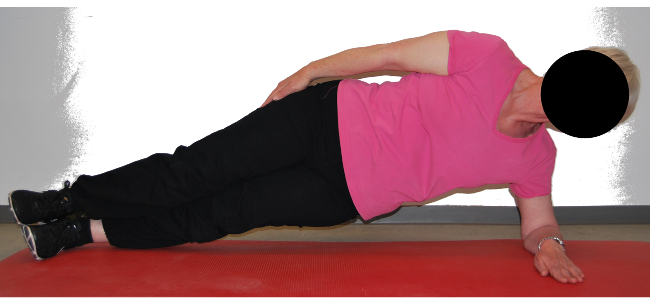


**D.** Lateral Plank: Side lying resting on one elbow positioned below the shoulder. The body is raised

to form a straight line, with resting point at the lateral aspect of the foot and elbow. The body is kept straight in the frontal plane. The exercise is repeated bilaterally.

**Progression:** When the participant is able to perform the exercise without distinct exertion and show control of the trunk, the participant is progressed to next level.


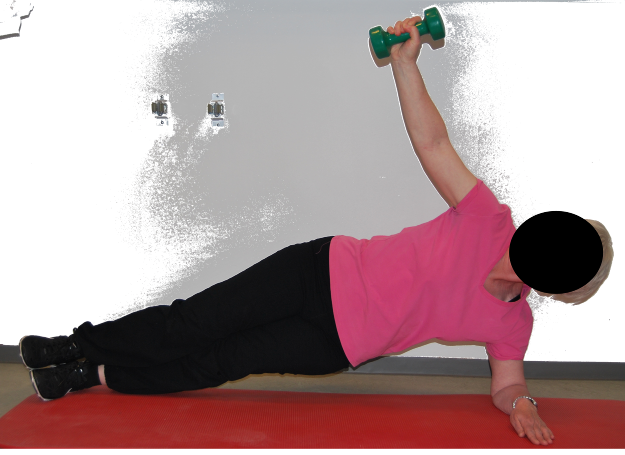
**E.** Lateral Plank with arm movement, progression with dumbbell: Starting position as in **D**. When the body is raised, the arm is raised to vertical and lowered in front of the body (shoulder adduction) and back to vertical. Direction of arm movement is changed regularly to ensure maximum challenge of the task. If it is too easy, a dumbbell can be used.

**Progression:** When the participant is able to perform the arm movement slowly and controlled with good control of the trunk, without distinct exertion, the progress to next level.

**F.** Lateral Plank with jumping jack movement, further progression v. dumbbell/Swiss ball: starting position as in **D**. Top leg and arm are abducted and adducted simultaneously. The dumbbell may be used as in **E**. **Progression:** If further progression is needed a Swiss ball can be placed under the elbow to make an unstable base of support.

**2.2 Pelvis/hip stability**

**Purpose:** To maintain hip and trunk stability during dynamic movements with external load or unstable base of support.

**Focus:** The pelvis must be lifted to the same height each time. It is important that the pelvis is kept from tilting in the frontal and transverse planes; the participant should aim to control the pelvic movement and position throughout the exercises. At all levels, good knee control is emphasised; the knee is kept approximately one hip width apart without hyperextension or excessive flexion.

**Repetitions**: 2-3×6-8. In **E-F** 2-3×6-8 on both sides

**Progression:** The exercises are progressed from level **A (easiest)** to **F (most difficult)**.


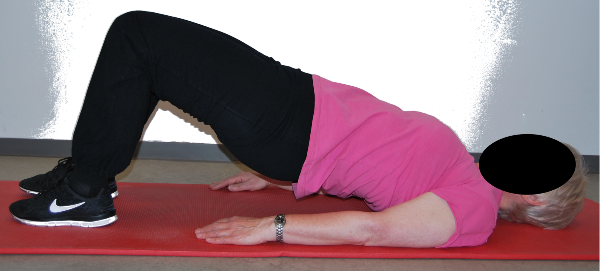
**A**. Supine pelvic lift: Supine with hips and knees flexed and the feet flat on the floor, approximately one hip width apart. Arms placed along the side of the body. The Pelvis is steadily and controlled lifted up and down again.

**Progression**: When the participant is able to perform the exercise without exertion and keep the pelvis stable during movement, progress to the next level.


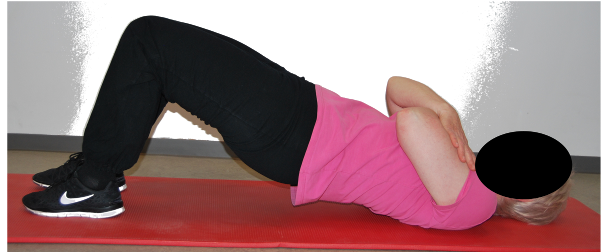
**B.** Pelvis lift as in **A**: Arms are crossed over the chest to decrease the base of support.

**Progression:** When the participant is able to perform the exercise without exertion and keep the pelvis stable during movement, progress to the next level.


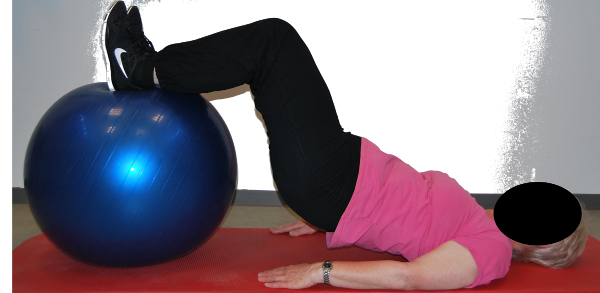
**C**. Pelvis lift with both feet on a Swiss ball: Supine with hips and knees flexed and heels placed on a Swiss ball. Arms placed along the side (if relevant, a bit abducted). The Pelvis is steadily and controlled lifted

up and down with no or minimal ball movement.

**Progression:** When the participant is able to lift the pelvis with no rotation or tilt, the knee flexed, without ball movement and no distinct exertion, progress to the next level.

**D**. Pelvis lift as in **C**., except for the arms, that are flexed at the elbows (only the upper arm touching the floor) to reduce base of support (can be further progressed by crossing arms across the chest before moving to level **E**).

**Progression:** When the participant is able to lift the pelvis while maintaining it stable and keeping the knee flexed, without ball movement and no distinct exertion, progress to the next level.


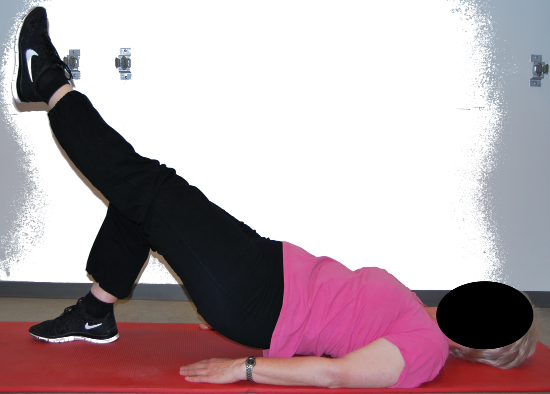
**E**. One-legged Pelvic lift: The exercise is performed as in

**A** or **B**., except this time only one leg is used for support. The other leg is held in level with the other. The exercise

is repeated on both sides.

**Progression:** When the participant is able to lift the pelvis with no rotation or tilt, and no distinct exertion, progress

to next level.


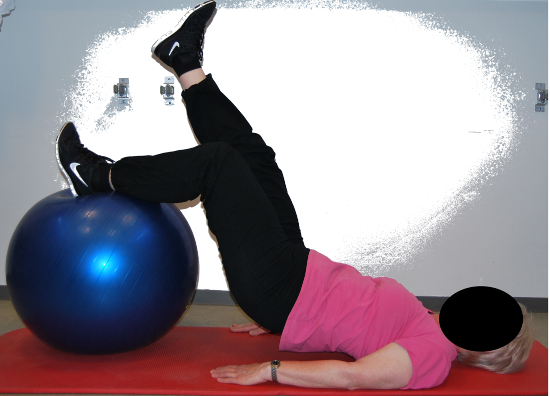


**F**. One-legged Pelvic lift whit Swiss ball: The exercise is performed as in **C**., except this time only one leg is used for support on the ball. The other leg is held above the Swiss ball. The exercise is repeated with both sides.

Further progression: Reducing the arm support (e.g. elbow flexion as in **D** or arms crossed across chest as in **B**.

**2.3. Gluteus medius strengthening**

**Purpose:** To strengthen the hip abductors.

**Focus:** From **A-D** the starting position is important: Side lying, the upper body leaning slightly forwards and the knees bent to 90 degrees flexion. When the knees are drawn apart it is important that the upper body is kept steady; if not, the exercise will not effectively target the gluteus medius. In **A-D** the movement is done in two parts: First, with feet held together the hips are maximally externally rotated (by separating the knees). When maximal external hip rotation is reached the heels are drawn apart in a vertical movement.

**Repetitions**: 2-3×6-8.

**Progression:** The exercises are progressed from level **A (easiest)** to **F (most difficult)**. All exercises are repeated on both sides.


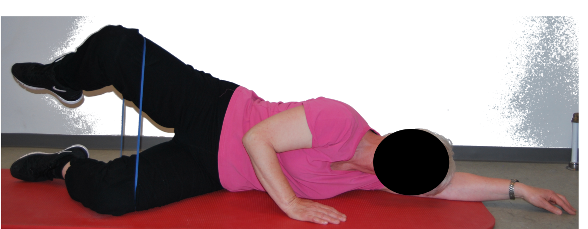


**A**. Side lying, knees in 90 degrees flexion, rubber band (light resistance) placed around the thighs, just proximal to the knees: Without moving the trunk or feet the knees are drawn apart (external hip). When maximal external hip rotation is reached the heels are drawn apart. After this the heels are drawn back together, followed by the knees.

**Progression:** When the participant is able to perform the maximal number of repetitions without exertion and no rotation of the pelvis, the progress to next level.

**B**. As in **A**, but with heavy resistance rubber band.

**Progression:** When the participant is able to perform the maximal number of repetitions without exertion and no rotation of the pelvis, the progress to next level.


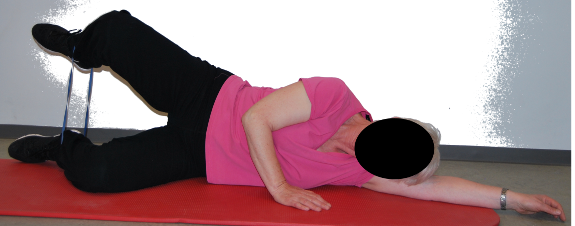
**C**. As in **A**, but with light resistance rubber band placed around the ankles.

**Progression:** When the participant is able to perform

the maximal number of repetitions without exertion

and no rotation of the pelvis, the progress to next level.

**D**. As in **C**, but with heavy resistance rubber band.

**Progression:** When the participant is able to perform the maximal number of repetitions without exertion and no rotation of the pelvis, the progress to next level.


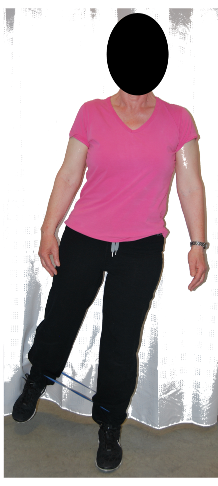
**E**. Standing Hip abduction/extension: Standing on one leg with light resistance rubber band around the ankles. With the pelvis and upper body in neutral position the hip of the unsupported leg is moved backwards and outwards (abduction + extension).

**Progression:** When the participant is able to perform the max repetitions without distinct exertion and can perform the abduction without additional movement of the hip and control of the trunk the progress to next level.

**F**. Same as **A,** but with heavy resistance rubber bands around the ankles.

**2.4 Knee control and stability**

**2.4.1. Knee end-extension control**

**Focus:** Good knee control throughout knee range of motion of each exercise is important. This is defined as alignment of the knee above the second toe. When the end-extension exercises are performed it is important to avoid hyper-extension of the knee. The goal for this exercise is to make the participant able to stop the knee from hyper-extending during movement.


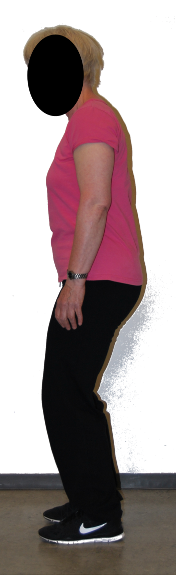
**Time**: 1-2 minutes. In **C** 1-2 minutes on both legs.

**Progression:** The exercises are progressed from level **A (easiest)** to **C (most difficult)**.

**A**. Fast knee end-extensions: Standing and weight bearing position with slight knee flexion (15-20 degrees). Fast (1-2 movement cycles/second) and controlled knee extensions of the knee are performed. The motion takes place within the last 15-20 degrees of knee extension.

In the beginning, the exercise is done with body weight equally placed on both feet. After this, while the movement speed is kept, weight is transferred from one leg to the other. When this is implemented with good control one-legged exercise can be added.


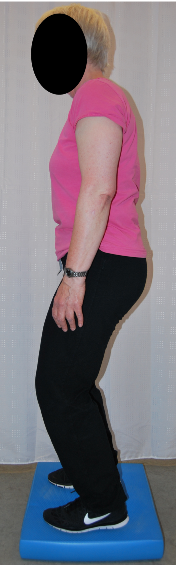
**Progression:** When the participant is able to maintain a regular rhythm of the continuous knee flexion and extension movement pattern without hyper-extension the progress to next level.

**B**. Knee end-extension on a small and unstable base of support: Same as in **A**, but

Standing on a rubber foam block - initially under both feet, but as in **A** progression to

one-legged exercise can be used.

**Progression:** When the participant is able to maintain a regular rhythm of the continuous

knee end-extension movement pattern without hyper-extension the progress to next level.

**
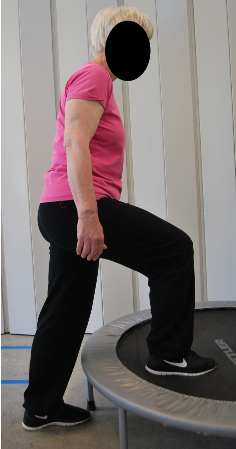
**

**C.** Standing with one leg on a trampoline. All support is shifted to the leg on the trampoline and a pumping movement is performed.

**2.4.2 Lunges**

**Focus:** Good knee control throughout the exercise is emphasised. This is defined as alignment of the knee over the second toe, to practice movement with good varus/valgus control. The purpose of this is exercise is to gain functional strength and teach the participant to control the knee during high load movements. The trunk should be upright with the back as straight as possible.

**Time**: 1-2 minutes

**Progression:** The exercises are progressed from level **A (easiest)** to **C (most difficult)**.


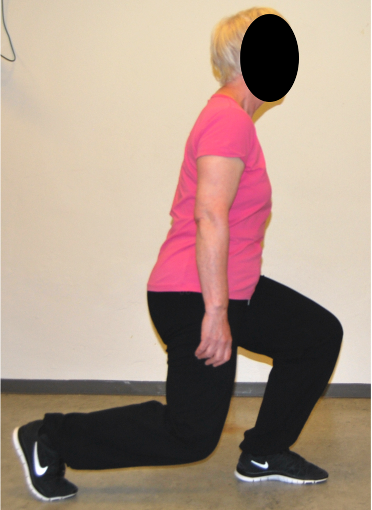


**A**. Lunges with variable step lengths: The leg is slowly and controlled moved forward to match the participant normal step length. When the foot is placed on the ground a controlled flexion of the knee is done to approximately 90 degrees. Then the leading knee is extended and the participant pushes back to the starting position. When this is done with good control, the step length is increased.

**Progression:** When the participant is able to perform a long lunge on both legs with good control, push straight back, with the centre of masse placed over the tailing limb, and with no pain or safe or acceptable pain intensity, the progress to next level.

**B**. Lunges whit focus on knee control during landing and takeoff of the foot: Performed as in **A**. but now the push off is done with a greater force.

**Progression:** When the participant is able to do the push off with no, safe or acceptable pain intensity, and land with good balance, the exercise is progressed to the next level.

**C**. Lunge walking: as in **A**., except now in stead of pushing back to the starting position, a similar movement with the trailing limb is done, resulting in a lunge walk. When this is done with good control the step lengths and knee flexion is increased.

**2.5. Strengthening exercise for the knee**

**2.5.1. Leg press or ball on the wall**

**Focus:** Good knee control throughout the exercise is emphasised. This is defined as alignment of the knee over the second toe. Feet are positioned approximately one hip width apart. In **B**. and **C**. it is important that the back is straight and that the knees are aligned over the feet. The knee flexion should happen in a way that makes the trunk move vertically up and down.

**Repetitions**: 2-3×8-10, if it is possible for the participant to take more than 10 repetitions per set, the exercise is too easy and more external weight should be applied.

**Progression:** The exercises are progressed from level **A (easiest)** to **C (most difficult)**.


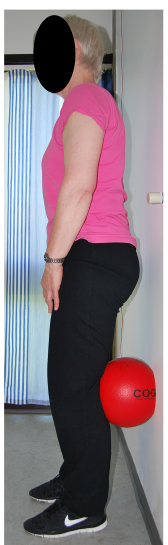


**A**. Small Swiss ball between the popliteal fossa and the wall. The ball is squeezed against the wall by performing a knee extension. Avoid hyperextension. The exercise is performed with one leg at the time.

**Progression:** When the participant is able to create a clear contraction of the m. vastus medialis obliquum, progress to next level.


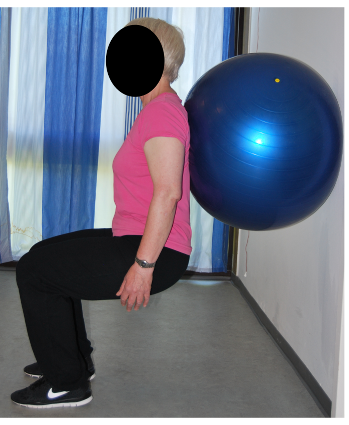


**B**. Squat with a Swiss ball between the back and a wall. The feet are placed a bit from the wall and a knee flexion to 90 degrees is performed. The trunk is kept upright with a straight back during the movement.

**Progression:** When the participant is able to perform the maximal number of repetitions, with knees bent to 90 degrees each time, without exertion and no, safe or acceptable knee pain, the progress to next level.

**C**. Squat with ball and dumbbell/weight west: as in **B**., except a dumbbell is held in each hand or a weight west is put on (5-10 kilos).

**2.5.2. Step-ups with rubber band**


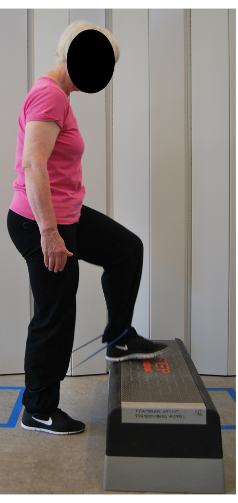
**Focus:** Knee control, defined as the knee aligned over 2^nd^ toe. It is important to minimize use of the vision to practice the participants ability to sense whether they have good knee control or not.

**Repetitions**: 3×1-2 minutes

**Progression:** The exercises are progressed from level **A (easiest)** to **E (most difficult)**.

**A**. Low step height, light rubber band, straight forward steps: From stride-standing one leg after the other is lifted up on the step, weight transferred to the step (step up), and backwards down again. This exercise is repeated until the performance is steady and with good knee control.

**Progression:** When the participant is able to perform the maximal number of repetitions, with no, safe or acceptable pain, the knee aligned over the 2^nd^ toe during weight transfer and step-ups, and no pelvis drop during movement the progress to next level.

**
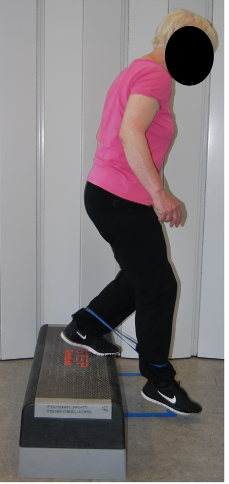
**

**B**. Low step height, tight rubber band, varied steps: Same as in **A**. except now the steps should be varied, e.g. lifting the leg higher or taking wider steps, picking up the pace or slowing it down, or stepping up and over (figure).

**Progression:** When the participant is able to perform the maximal number of repetitions, with no, safe, or acceptable pain, knee aligned over 2^nd^ toe, and no pelvis drop during movement the progress to next level.

**C**. Medium step height, light rubber band, varied steps: As in **B**. except the step is higher and a light rubber band is used.

**Progression:** When the participant is able to perform the maximal number of repetitions, with no, safe, or acceptable pain, knee aligned over 2^nd^ toe, and no pelvis drop during step-up the progress to next level.

**D**. Medium step height, tight rubber band, varied steps: As in **C**., but with tight rubber band.

**Progression:** When the participant is able to perform the maximal number of repetitions, with no, safe, or acceptable pain, knee aligned with 2^nd^ toe, and no pelvis drop during step-up the progress to next level.

**E**. High step height, tight rubber band, varied steps: As in **D**., except the step height is higher. If this is too difficult but **D**. is to easy a light rubber band can be used. **2.6 Functional exercises (Level and Stair walking)**

**Focus:** Good knee alignment and a controlled and stable gait. When this is acquired the participant should be encouraged to walk without looking down and sense whether there is good knee alignment and control or not. Experiences acquired from exercises 2.1-2.5 is emphasised to the participants during walking and stair walking.

A “good” walking pattern includes looking straight ahead, natural rotation of the trunk with arms swinging freely. Trendelenburg and/or other compensatory walking patterns should be avoided. With each stride the goal is to have symmetry and normal clearance of the foot. The foot should be placed on the ground with heel first, and a nice roll over the foot to the greater toe at toe off. If relevant the participant should be encouraged to wear ergonomic shoes.

**Repetitions**: 5-10 minutes of continuous level and stair walking

**Progression:** The exercises are progressed from level **A (easiest)** to **B (most difficult)**.


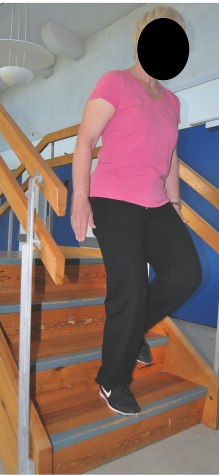


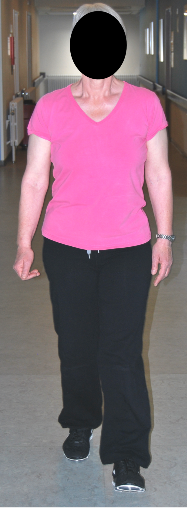
**A**. Walk with a “good” walking pattern and combine with staircase walking to simulate everyday movements. Focus should be on performing these everyday movements as efficiently as possible. The physiotherapist should instruct each participant individually of his or her focus areas – recalling acquired skills and experiences from exercises 2.1 to 2.5.

**Progression:** When the participant is able to perform the physiotherapist’s individual instruction with ease the progress to next level.

**B**. Is in **A.** except external resistance is added. This could be increased speed, a rubber band around the thighs, ankle weights, or weight west to increase difficulty and/or resistance. The physical therapist decides what external resistance is suitable for the individual participant.

**“Rescue exercise program”**

If a participant scores knee pain above 5 on a 0-10 numerical rating scale (NRS; 0= no pain, 10= excruciating pain) when attending for an exercise session, he/she is transferred to the rescue program.

The rescue program is a modified program consisting of an extended warm up (15-20 minutes), and exercises 2.1, 2.2, 2.3 (as described above), repeated twice with a 5 minute ergometer cycling in between.

The rescue program excludes any weight bearing activities to avoid unwarranted symptom provocation during a period of symptomatic flare-up.

**7. References**

(1) Roos EM, Roos HP, Lohmander LS, Ekdahl C, Beynnon BD. Knee Injury and Osteoarthritis Outcome Score (KOOS)--development of a self-administered outcome measure. J Orthop Sports Phys Ther 1998 Aug;28(2):88-96.

(2) Lund H, Sondergaard K, Zachariassen T, Christensen R, Bulow P, Henriksen M, et al. Learning effect of isokinetic measurements in healthy subjects, and reliability and comparability of Biodex and Lido dynamometers. Clin Physiol Funct Imaging 2005 Mar;25(2):75-82.

(3) Jaric S. Muscle strength testing: use of normalisation for body size. Sports Med 2002;32(10):615-31.

(4) Dobson F, Hinman RS, Roos EM, Abbott JH, Stratford P, Davis AM, et al. OARSI recommended performance-based tests to assess physical function in people diagnosed with hip or knee osteoarthritis. Osteoarthritis Cartilage 2013 Aug;21(8):1042-52.

(5) Enright PL, McBurnie MA, Bittner V, Tracy RP, McNamara R, Arnold A, et al. The 6-min walk test: a quick measure of functional status in elderly adults. Chest 2003 Feb;123(2):387-98.

(6) Enright PL. The six-minute walk test. Respir Care 2003 Aug;48(8):783-5.

(7) Kubassova O, Boesen M, Cimmino MA, Bliddal H. A computer-aided detection system for rheumatoid arthritis MRI data interpretation and quantification of synovial activity. European Journal of Radiology 2010;74(3):67-72.

(8) Riis RG, Gudbergsen H, Simonsen O, Henriksen M, Al-Mashkur N, Eld M, et al. The association between histological, macroscopic and magnetic resonance imaging assessed synovitis in end-stage knee osteoarthritis: a cross-sectional study. Osteoarthritis Cartilage 2016 Oct 11.

(9) Kubassova O, Boesen M, Peloschek P, Langs G, Cimmino MA, Bliddal H, et al. Quantifying Disease Activity and Damage by Imaging in Rheumatoid Arthritis and Osteoarthritis. Annals of the New York Academy of Sciences 2009 Feb 15;1154:207-38.

(10) Boesen M, Kubassova O, Cimmino MA, Ostergaard M, Taylor P, Danneskiold-Samsoe B, et al. Dynamic Contrast Enhanced MRI Can Monitor the Very Early Inflammatory Treatment Response upon Intra-Articular Steroid Injection in the Knee Joint: A Case Report with Review of the Literature. Arthritis 2011;2011:578252.

(11) Axelsen MB, Poggenborg RP, Stoltenberg M, Kubassova O, Boesen M, Horslev-Petersen K, et al. Reliability and responsiveness of dynamic contrast-enhanced magnetic resonance imaging in rheumatoid arthritis. Scand J Rheumatol 2013;42(2):115-22.

(12) Boesen M, Kubassova O, Bouert R, Axelsen M, Ostergaard M, Cimmino MA, et al. Correlation between computer-aided dynamic gadolinium-enhanced MRI assessment of inflammation and semi-quantitative synovitis and bone marrow oedema scores of the wrist in patients with rheumatoid arthritis-a cohort study. Rheumatology 2012;51(1):134-43.

(13) Ballegaard C, Riis RG, Bliddal H, Christensen R, Henriksen M, Bartels EM, et al. Knee pain and inflammation in the infrapatellar fat pad estimated by conventional and dynamic contrast-enhanced magnetic resonance imaging in obese patients with osteoarthritis: A cross-sectional study. Osteoarthritis and Cartilage 2014;22(7):933-40.

(14) Riis RG, Henriksen M, Klokker L, Bartholdy C, Ellegaard K, Bandak E, et al. The effects of intra-articular glucocorticoids and exercise on pain and synovitis assessed on static and dynamic magnetic resonance imaging in knee osteoarthritis: exploratory outcomes from a randomized controlled trial. Osteoarthritis Cartilage 2016 Oct 13.

(15) Riis RG, Gudbergsen H, Henriksen M, Ballegaard C, Bandak E, Rottger D, et al. Synovitis assessed on static and dynamic contrast-enhanced magnetic resonance imaging and its association with pain in knee osteoarthritis: A cross-sectional study. Eur J Radiol 2016 Jun;85(6):1099-108.
